# A Distinct Immune Signature Characterizes Cirrhosis in Ethiopian Individuals With Chronic HBV Infection

**DOI:** 10.1111/apt.70816

**Published:** 2026-07-01

**Authors:** Julia MacLeod, Fikadu G. Gudissa, Asiya Jeylan, Nega Berhe, Hailemichael Desalegn, Lasse Rossvoll, Asgeir Johannessen, Niklas K. Björkström, Dag Henrik Reikvam, Annika Niehrs

**Affiliations:** ^1^ Institute of Clinical Medicine University of Oslo Oslo Norway; ^2^ Department of Infectious Diseases Oslo University Hospital Oslo Norway; ^3^ Adama Comprehensive Specialized Hospital Medical College Adama Ethiopia; ^4^ Department of Infectious Diseases Vestfold Hospital Tønsberg Norway; ^5^ Aklilu Lemma Institute of Pathobiology Addis Ababa University Addis Ababa Ethiopia; ^6^ St. Paul's Hospital Millenium Medical College Addis Ababa Ethiopia; ^7^ Centre for Infectious Medicine, Department of Medicine Huddinge Karolinska Institutet, Karolinska University Hospital Stockholm Sweden

**Keywords:** Africa, cirrhosis, hepatitis B, immunophenotyping

## Abstract

**Background and Aims:**

The immune cell phenotype of African patients living with chronic hepatitis B virus (HBV) infection is rarely described, despite high infection rates. This study investigated the differences in immune cell phenotypes in four distinct clinical phases of disease in an Ethiopian cohort.

**Methods:**

Two hundred eighty‐seven patients with untreated HBV infection, and no human immunodeficiency virus and hepatitis C virus infection, were included at Adama Hospital, Ethiopia. Patients were categorized based on clinical parameters into the following groups: low‐replicative infection, “greyzone”, active hepatitis, and cirrhosis. Whole blood samples were cryopreserved using Cytodelics Whole Blood Cell Stabilizer and analysed using two high‐dimensional flow cytometry panels to define immune cell phenotypes.

**Results:**

Patients with cirrhosis displayed a distinct immunophenotype compared to other phases of chronic HBV infection with an increase in monocytes and a reduction in all lymphocytes alongside a shift from a mature to a naïve phenotype in NK cells, CD4^+^ T cells, and B cells. The emergence of activated CD38^+^HLA‐DR^+^ cells across lymphocyte lineages defined patients with cirrhosis and the frequency of these double positive cells correlated positively with the AST to Platelet Ratio Index used as a measure of cirrhosis severity.

**Conclusions:**

HBV‐associated cirrhosis in Ethiopia displayed a unique immunophenotype, in which the frequency of CD38^+^HLA‐DR^+^ NK cells and T cells correlated strongly with the severity of cirrhosis.

## Graphical abstract

Cirrhosis patients with untreated HBV in Ethiopia exhibit a distinct immune profile marked by depletion of NK cells, B cells, and T cells, alongside an expanded CD38^+^HLA‐DR^+^ CD8 T‐cell population.

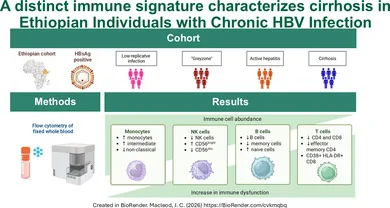

**Abbreviations**
ALT: alanine aminotransferase
APRI: AST to Platelet Ratio Index
AST: aspartate aminotransferase
CAID: cirrhosis‐associated immune dysfunction
FBC: full blood count
FNA: Fine needle aspirate
HBsAg: hepatitis B surface antigen
HBV: hepatitis B virus
HCV: hepatitis C virus
HDV: hepatitis D virus
HIV: human immunodeficiency virus
MAIT: mucosal‐associated invariant T cells
PBMC: peripheral blood mononuclear cells
TEMRA: terminal effector memory cells re‐expressing CD45RA
WHO: World Health Organization

## 1 Introduction

In 2022, 771,000 new hepatitis B virus (HBV) infections and 272,000 HBV‐related deaths were registered in the World Health Organization (WHO) African region [1]. In Ethiopia, the prevalence of chronic HBV infection has been estimated to be between 6.0% and 6.9% [2, 3] which translates to a total of 7 million people living with HBV [2].

Current standard treatments for chronic HBV infection suppress viral replication and reduce the risk of liver complications, specifically hepatocellular carcinoma and cirrhosis. However, not all patients require medicine to prevent disease progression. To make precise decisions on indication for antiviral therapy, in‐depth knowledge of the immunopathogenesis of HBV infection is essential.

Immune cells have been described as altered across distinct clinical phases of HBV infection, with T cells having a pivotal role in determining the course of both acute and chronic HBV infection [4]. CD8^+^ T cells have previously been described as expressing markers of immune cell exhaustion with reduced cytotoxic potential in chronic HBV [5]. However, more recent research studies observe that these cells display varying degrees of functionality with HBV specific CD8^+^ T cells retaining effector function in patients with endogenous control of infection [6, 7]. Additionally, the liver microenvironment heavily influences the extent of CD8^+^ T cell dysfunction [8, 9]. Although less studied, CD4^+^ T cells also play an important role in HBV infection with HBV‐specific CD4^+^ T cells being increased in frequency in patients who achieve functional cure (defined as a loss of HBsAg) [10]. Similar to CD8^+^ T cells, CD4^+^ T cells within the liver can influence the degree of T cell dysfunction via licencing of myeloid cells [11]. Mucosal associated invariant T cells (MAIT) are reduced across all stages of disease compared with healthy controls [12]. Evidence for dysfunction in B cells during chronic HBV infection is conflicting. Atypical memory B cells are expanded in patients with chronic hepatitis B and express multiple inhibitory markers that impair their functions [13], and this fraction of HBV specific atypical memory B cells retracts when patients achieve functional cure [14]. However transcriptional profiles reveal that B cells in chronic immune active HBV infection have an activated phenotype in both the liver and peripheral blood [15].

Importantly, most of these immunological studies have been performed in Asian or European patients, with African cohorts being largely absent from the literature despite the high disease burden in the region. Improved knowledge of the specific immunopathology in African patients is important, as disease modifying factors such as environmental exposures, co‐infections, and genetic background differ from other parts of the world [16, 17, 18].

In order to perform the immunophenotypic assessment in this study, Cytodelics Whole Blood Cell Stabilizer was used to cryopreserve whole blood at the hepatitis B clinic in Ethiopia, one of several newer cryopreservative reagents with results comparable to standard freezing methods for peripheral blood mononuclear cells (PBMCs) [19]. This allowed for assessment of all immune cell compartments and inclusion of in‐depth analysis of T cells and natural killer (NK) cells across various stages of clinical disease ranging from low‐replicative infection to advanced liver disease in a well‐characterized cohort of Ethiopians living with untreated HBV [20].

## 2 Patients and Methods

### 2.1 Study Population

Patients were recruited at the Hepatitis Clinic at Adama Comprehensive Specialized Hospital, Ethiopia. HBsAg‐positive individuals aged 18 years or above, with a negative human immunodeficiency virus (HIV) and anti‐hepatitis C virus (HCV) test, were included. Detailed medical history and clinical parameters were recorded. Liver stiffness measurement (LSM) was performed using transient elastography (Fibroscan, Echosens, France) in 125 patients, and ultrasound in 170, with results used to aid staging of clinical disease. All participants provided written informed consent in their native language. The study was approved by the National Research Ethics Review Committee in Ethiopia (3.10/829/07) and the Regional Committees for Research Ethics in Norway (2014/1146) and all research was conducted in accordance with both the Declarations of Helsinki and Istanbul. No blinding was performed, and no statistical tests were used to determine patient numbers included in the study. The number of samples included within the study was based on sample availability and quality of the sample.

### 2.2 Haematological, Biochemical and Virological Testing

The following laboratory diagnostics were performed for each patient: leukocyte count, aspartate aminotransferase (AST), alanine aminotransferase (ALT), bilirubin and creatinine. Rapid diagnostic tests were performed for HIV antibody/antigen, HCV antibody and HBsAg initially using HBsAg‐2 (Abbott Diagnostics, IL, USA), thereafter based on local availability [20]. Anti‐HDV antibodies were tested using an in‐house pan‐genotypic ELISA assay in Heidelberg, Germany, and confirmed using a commercial ELISA assay on all samples (ETI‐AB‐DELTAK‐2, Diasorin, Saluggia, Italy). The Xpert HBV Viral Load kit (Cepheid, CA, USA) was used for measuring HBV DNA levels. Quantitative HBsAg (qHBsAg) was tested using the Elecsys HBsAg II Quant assay (Roche Diagnostics GmbH, Mannheim, Germany) on the Roche Cobas e801 platform according to the manufacturer's instructions [21]. AST to Platelet Ratio Index (APRI) was calculated and a cutoff of < 0.28 was used to exclude significant fibrosis/cirrhosis based on a meta‐analysis of African HBV patients [22].

### 2.3 Patient Categories

Patients were grouped into the following categories: low‐replicative infection, “greyzone”, active hepatitis, and cirrhosis using the available clinical and biochemical information (Table 1). HBeAg was not available to characterize patients. Clinical groups were defined based on the 2017 EASL recommendations available at the time of patient inclusion [23]. 1000 patient samples were collected. Three hundred eighty‐two were excluded in the present study as they did not fit into the defined clinical groups, 37 were excluded due to previous treatment for HBV, 12 were excluded due to missing or inconclusive HCV or HIV test, 91 patients were excluded due to pregnancy, and 201 samples were excluded due to poor sample quality (defined as less than 10,000 cells in the CD45^+^ gate after neutrophil exclusion), leaving a total of 287 included samples in the present study (Table S1).

**TABLE 1:** Definitions of patient categories.

Low‐replicative infection	“Greyzone”	Active hepatitis	Cirrhosis
ALT ≤ 40 U/L *AND* HBV DNA ≤ 2000 IU/mL *AND* no fibrosis/cirrhosis: APRI < 0.28 No liver disease on ultrasound (if performed) LSM < 8.0 kPa (if performed)	ALT ≤ 40 U/L *AND* HBV DNA > 2000 IU/mL *AND* no fibrosis/cirrhosis: APRI < 0.28 No liver disease on ultrasound (if performed) LSM < 8.0 kPa (if performed)	ALT > 40 U/L *AND* HBV DNA > 2000 IU/mL *AND* No cirrhosis (as per cirrhosis category)	Clinical signs (ascites) *OR* Use of furosemide/spironolactone for previously diagnosed ascites *OR* Ultrasound findings (cirrhosis/ascites/portal hypertension) *OR* LSM > 11.7 kPa (not if ALT > 200 U/L)

### 2.4 Immune Cell Preservation and Staining

Blood‐derived immune cells were preserved using Cytodelics Whole Blood Cell Stabilizer (Cytodelics AB, Sweden) according to the manufacturer's instructions. In detail, whole blood was collected in EDTA tubes, and 0.6 mL blood was added to 0.6 mL Cytodelics Whole Blood Cell Stabilizer. The vial was inverted multiple times, incubated at room temperature for a minimum of 10 min, and frozen at −20°C locally for up to 2 months before being shipped on dry ice to a central biobank facility in Addis Ababa for long‐term storage at −80°C. Samples were subsequently shipped on dry ice to Scandinavia for in‐depth immune cell analysis. After being thawed, samples were washed with FACS buffer (PBS supplemented with 2% FBS (Merck) and 2 mM EDTA (Thermo Fisher Scientific)) until red blood cells were removed and subsequently stained with either CD45‐AF700 (Biolegend), CD45‐BUV805 (BD Biosciences), or both. Cells were washed with FACS buffer, and three patients with different CD45‐barcode were combined for subsequent staining with primary antibodies overnight [24]. Two flow cytometry panels were used, panel 1 focusing on broad immune subset phenotyping whilst panel 2 included markers of immune cell activation and exhaustion. A detailed overview of all antibodies used within the study is displayed in Table S2. Cells were washed and fixed using the FoxP3 Transcription Factor Staining Kit (Invitrogen), and Accucount blank particle counting beads (Spherotech) were added before samples were analysed on the BD FACS Symphony A5 (BD Biosciences) flow cytometer.

### 2.5 Data Analysis

FCS files were analysed using FlowJo (v10.10.0) and manual gating (Figure S1). Cluster and Uniform Manifold Approximation and Projection (UMAP) analysis was performed using the UMAP (v4.0.4) and Phenograph (v4.0.5) plugins with a K‐nearest neighbour (k‐NN) value of 90 for CD8 and CD4 T cells and k‐NN of 30 for NK cells. Four patients randomly selected from each clinical category but run on the same day to limit batch effect, were included for both CD8^+^, CD4^+^ T cells and NK cell cluster analysis. Heatmap expression and visualisation of these cell subgroups was performed in R Studio (v2024.12.1+563) using flowcore (v2.20.0), pheatmap (v1.0.13), tidyverse (v.2.0.0) and dplyr (v1.1.4) packages.

Statistical analysis was performed using Prism (v10, GraphPad). Differences in cell frequencies and cell counts between groups were measured using a non‐parametric Kruskal–Wallis test with post‐analysis Dunn's test. CHI^2^ test was used for patient sex. Significant differences are displayed as *p < 0.05, **p < 0.01, ***p < 0.001, ****p < 0.0001.

Correlation analysis was performed on the cirrhosis group using Spearman's rank correlation in Prism (v10, GraphPad) comparing both APRI score and the frequency of HLA‐DR^+^CD38^+^ NK cells, CD4^+^ T cells, and CD8^+^ T cells.

## 3 Results

### 3.1 Patient Characteristics

The median age of the herein characterized HBV‐infected cohort was 30 years (interquartile range 25–38) and 135/287 (47%) were female. By definition, clinical groups were different with regards to ALT, HBV DNA, APRI, LSM and platelets (Tables 2 and S1). Male sex was more prominent within the cirrhosis group (49/63, 78%), while the low‐replicative infection group was predominantly female (74/118, 63%). Patients within the cirrhosis group were significantly older with lower platelets and higher HBV DNA. The “greyzone” group had a higher qHBsAg compared with both the low‐replicative infection and cirrhosis group (Table 2, Figure S2).

**TABLE 2:** Patient cohort characteristics according to disease stage.

	Low‐replicative infection *N* = 118	“Greyzone” *N* = 51	Active hepatitis *N* = 55	Cirrhosis *N* = 63	*p*
Male sex	44 (37%)	22 (43%)	37 (67%)	49 (78%)	< 0.0001
Age	30 [25–38]	28 [25–34]	27 [22–32]	32 [26–40]	0.0013
Platelets (10^9^/L)	262 [230–303]	262 [219–296]	215 [157–264]	114 [72–201]	n/a
ALT (IU/L)	17 [14–22]	22 [17–26]	70 [50–90]	43 [31–63]	n/a
APRI	0.20 [0.16–0.24]	0.21 [0.17–0.26]	0.68 [0.44–1.06]	1.07 [0.58–2.50]	n/a
HBV DNA (IU/mL)	230 [45–799]	7280 [3515–25,050]	119,000 [13,050–9,600,000]	140,000 [1065–5,790,000]	n/a
LSM (kPa)	4.6 [4–4.9]	5.2 [4.8–5.7]	5.8 [4.8–7.3]	12.1 [6.1–17.3]	n/a
qHbsAg (IU/mL)	3309 [691–10,397]	8050 [2792–15,552]	5380 [2599–13,897]	3023 [798–6079]	0.0006

*Note:* Values presented as median [interquartile range] except sex presented as number (%). *p* values calculated using Chi^2^ test for sex and Kruskall–Wallis test for all other values. *p* values displayed as n/a were not calculated due to their inclusion in the definition of the groups.

### 3.2 Monocyte Frequency Increases in Later Stages of HBV Infection

**FIGURE 1:**
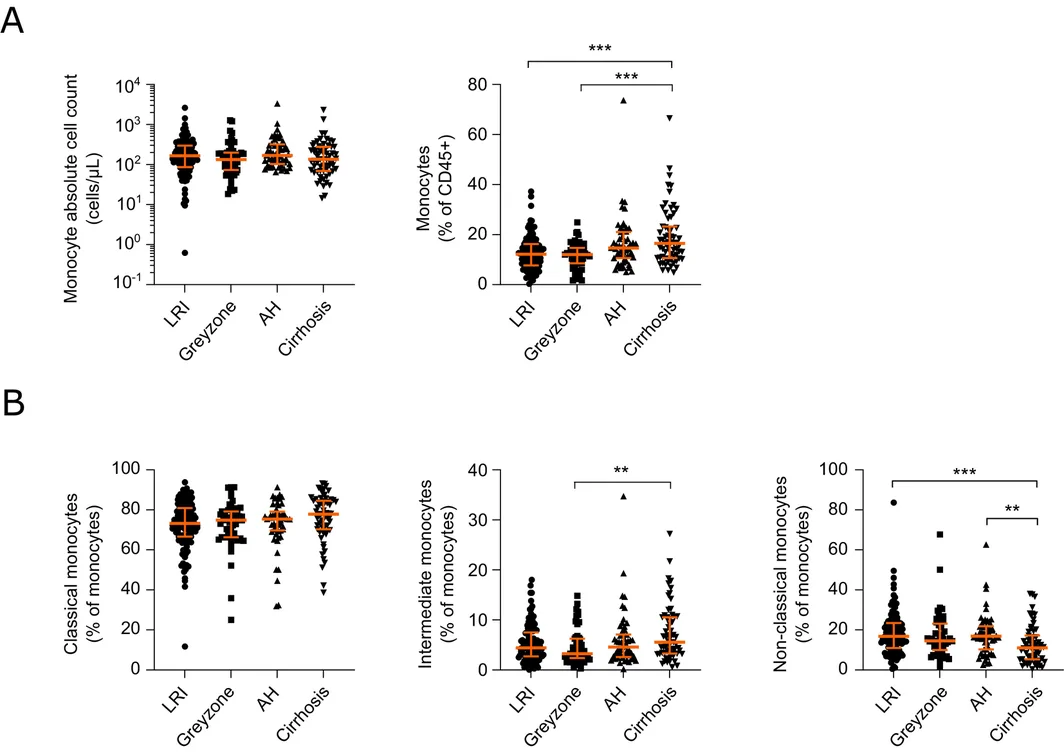
Monocyte frequencies change in distinct stages of chronic HBV infection. (A) Monocyte cell count (cells/μL) (left). Monocytes as a percentage of lymphocytes (CD45^+^ cells) (right). (B) Frequency of classical (left), intermediate (middle), and non‐classical (right) monocytes. Each dot represents one donor (*n* = 118 low‐replicative infection, *n* = 51 “greyzone”, *n* = 55 active hepatitis, *n* = 63 cirrhosis). Line represents the median; error bars represent interquartile range. Statistical differences were assessed using Kruskal–Wallis test with post hoc Dunn's test *p < 0.05; **p < 0.01; and ***p < 0.001. Only significant differences are displayed. AH, acute hepatitis; LRI, low‐replicative infection.

To better understand how disease progression in HBV infection influences immune cell characteristics, the phenotype of immune cells within the respective clinical groups was assessed. Monocytes were defined as CD3^−^CD19^−^CD56^−^CD141^−^CD1c^−^HLA‐DR^+^ thereby excluding dendritic cells, then classified as classical (CD14^+^CD16^−^), intermediate (CD14^+^CD16^+^), or non‐classical (CD14^lo^CD16^+^) (Figure S1 & Table S3). The relative frequency of all monocytes was significantly higher in cirrhosis compared with both low‐replicative infection and “greyzone” (median frequency 16.5% vs. 12.2% and 12.0% respectively), while no differences were observed for the absolute monocyte counts (Figure 1A and Table S4). Analysis of the individual monocyte subsets displayed an increase of intermediate monocytes in cirrhosis compared with the “greyzone” (median frequency 5.6% vs. 3.2%) and decreased non‐classical monocytes compared with both low‐replicative infection and active hepatitis (median frequency 11.0% vs. 16.8% and 16.9% respectively) (Figure 1B). Although not statistically different, the classical and intermediate monocyte population showed a tendency towards being increased in the cirrhosis group. No difference was observed in HLA‐DR expression across the monocyte subtypes (data not shown).

In contrast to altered frequencies within monocyte populations, we did not observe significant differences in either CD141^+^ or CD1c^+^ dendritic cell population across the different clinical groups (data not shown).

### 3.3 CD56^bright^ NK Cells Are Enriched in HBV‐Associated Cirrhosis

**FIGURE 2:**
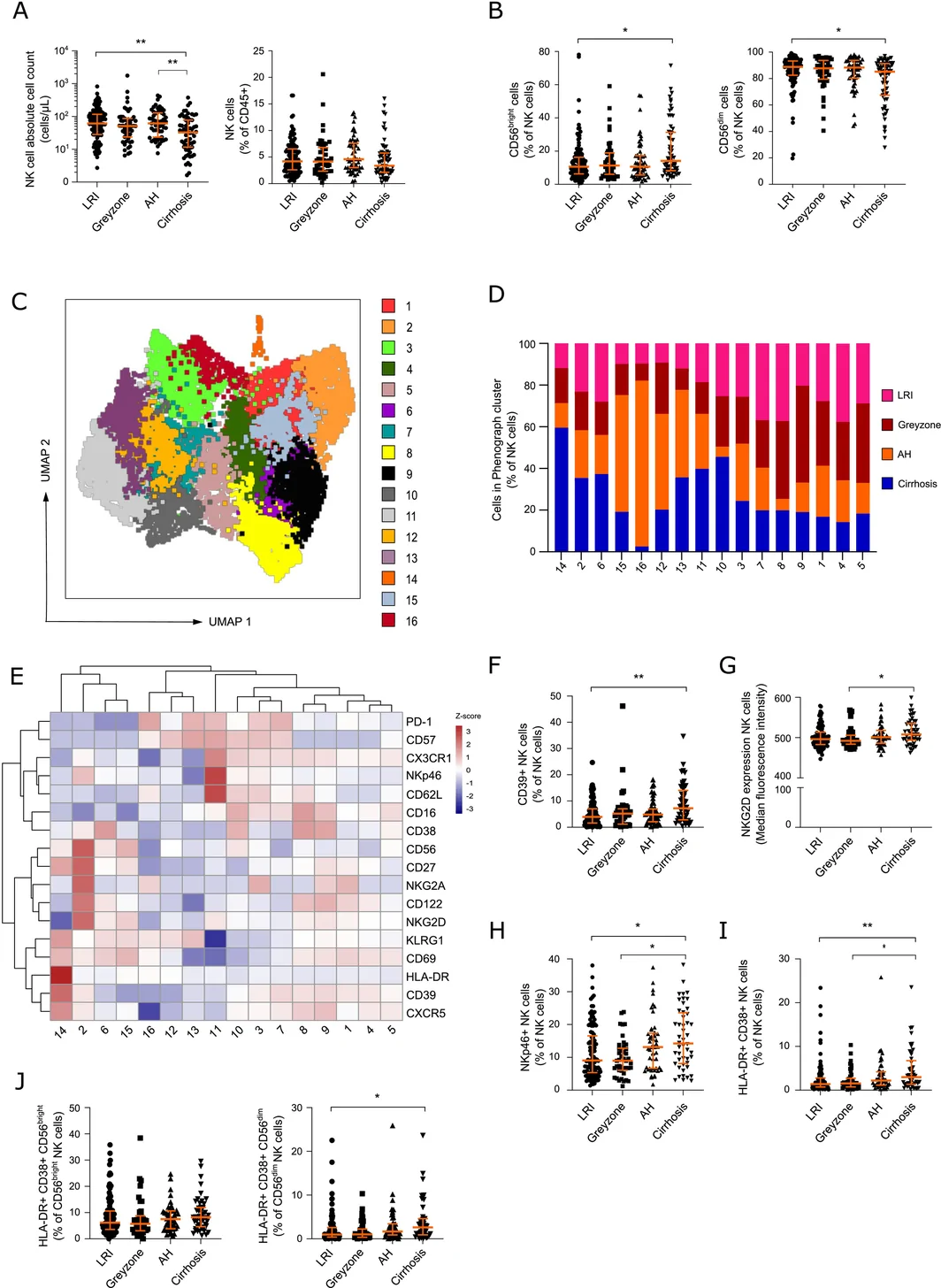
NK cell maturation and phenotype is influenced by clinical stages of chronic HBV. (A) NK cell counts (cells/μL) (left) and as percentage of lymphocytes (CD45^+^ cells) (right). (B) CD56^bright^ (left) and CD56^dim^ (right) as percentage of total NK cells. Each dot represents one donor (*n* = 118 low‐replicative infection, *n* = 51 “greyzone”, *n* = 55 active hepatitis *n* = 63 cirrhosis). Line represents the median; error bars represent interquartile range. (C) UMAP of NK cells. 590 NK cells from each of 4 patients from each clinical group (*n* = 16) were concatenated. Phenograph analysis showing 16 NK cell clusters overlaid onto the UMAP analysis. (D) Phenograph clusters displayed as stacked bar graph showing distribution of each cluster in each clinical group. (E) Heatmap displaying each individual NK cell Phenograph cluster (*x*‐axis) with median fluorescence intensity *z*‐score for indicated markers (*y*‐axis). Hierarchical clustering was done using flowcore (v2.20.0), pheatmap (v1.0.13), tidyverse (v.2.0.0) and dplyr (v1.1.4) packages in R studio. (F) CD39^+^ NK cell frequency of total NK cells in four clinical groups. (G) NKG2D expression in NK cells as MFI. (H) NKp46^+^ NK cells. (I) HLA‐DR^+^ CD38^+^ NK cells. (J) Frequencies of HLA‐DR^+^ CD38^+^ NK^bright^ cells (left) and NK^dim^ cells (right). Each dot represents one donor (*n* = 109 low‐replicative infection, *n* = 42 “greyzone”, *n* = 49 active hepatitis *n* = 49 cirrhosis). Line represents the median; error bars represent interquartile range. Statistical differences were assessed using Kruskal–Wallis test with post hoc Dunn's test *p < 0.05; **p < 0.01; and ***p < 0.001. Only significant differences are displayed. AH, acute hepatitis; LRI, low‐replicative infection; MFI, median fluorescence intensity; NK, natural killer; UMAP, uniform manifold dimensionality reduction.

NK cells (CD45^+^CD3^−^CD19^−^CD56^+^) have been described as having conflicting roles within HBV infection; they can contribute to infection control as well as liver cirrhosis development [25]. Initial analysis showed a lower absolute NK cell count in cirrhosis compared with the low‐replicative infection and active hepatitis (32.5 vs. 61.2 cells/μL and 61.5 cells/μL respectively) but no differences in NK cell frequencies (Figure 2A and Table S4). The distribution of NK cell subsets was altered in patients with cirrhosis with a significant increase in CD56^bright^ NK cells compared to low‐replicative infection (median frequency 14.1% vs. 10.6%) and a reciprocal decrease in CD56^dim^ NK cells (median frequency 85.2% vs. 88.8%) (Figure 2B), indicating that in cirrhosis, cytotoxic effector CD56^dim^ NK cells are diminished, while less mature CD56^bright^ NK cells are more frequent.

To characterize the NK cell phenotype in greater detail, unsupervised clustering was used to identify distinct NK cell clusters within each clinical group (Figure S3), revealing 16 NK cell clusters (Figure 2C). Cluster 14 was predominant in cirrhosis and displayed a phenotype with high expression of HLA‐DR, CD39, CXCR5, CD69, and KLRG1 (Figure 2D,E). This is reflected in the overall frequencies, with a significant increase in CD39 expression in cirrhosis (Figure 2F). Cluster 10 was also more prevalent in the cirrhosis group and displayed increased expression of CD16 and CD38, as well as CD57, suggestive of an activated, well‐differentiated NK cell cluster in the CD56^dim^ cell compartment [26]. NKG2D expression was increased in cirrhosis compared with “greyzone” (median fluorescence intensity 508 vs. 492) (Figure 2G), with increased expression also found in cluster 2, a cluster with a higher percentage of cells from the cirrhosis group. Additionally, expression of NKp46 was significantly increased in NK cells in cirrhosis compared with both low‐replicative infection and “greyzone” (median frequency 14.2% vs. 9.04% and 8.95%) (Figure 2H), and cells predominantly from cirrhosis in cluster 11 notably had high expression of NKp46 (Figure 2D,E).

Next, the presence of CD38^+^ and HLA‐DR^+^ NK cells was investigated as they have been implicated in the progression of other chronic viral infections [27] and are increased in acute HBV and HDV infection [28]. There was a significant increase in CD38^+^ HLA‐DR^+^ NK cells in cirrhosis compared to low‐replicative infection and “greyzone” (median frequency 2.97% vs. 1.39% and 1.54% respectively) (Figure 2I), which was mainly driven by the CD56^dim^ NK cells (Figure 2J).

### 3.4 A Shift From Memory to Naïve B Cell Subsets Is Observed Within HBV‐Associated Cirrhosis

B cell populations were characterized by first defining plasmablasts and B cell precursors (CD3^−^CD19^+^CD20^−^CD38^+^) (Figure S1) and subsequently gating on CD19^+^CD20^+^ B cells. Significantly lower B cell counts were observed in cirrhosis compared with both low‐replicative infection and “greyzone” (median count 70.3 vs. 114.0 cells/μL and 121.6 cells/μL respectively) (Figure S4A and Table S4). B cells were then gated into switched memory (CD27^+^ IgD^−^), non‐switched memory (CD27^+^ IgD^+^), naïve (CD27^−^ IgD^+^) and atypical memory B cells (CD27^−^ IgD^−^) based on CD27 and IgD expression. Frequencies of non‐switched memory and atypical memory B cells did not display significant changes (data not shown). In contrast, a significant reduction of switched memory B cells in cirrhosis compared to other clinical groups (median frequency 11.4% vs. 18.2% low‐replicative infection, 17.6% “greyzone” and 16.5% active hepatitis) and a reciprocal increase in naïve B cells compared to low‐replicative infection and “greyzone” (median frequency 66.2% vs. 56.9% and 54.7% respectively) was observed (Figure S4B), suggesting a decreased ability to rapidly respond to recurring antigen stimuli.

### 3.5 Frequencies of CD4 ^+^ T Cell Populations Change With Progression of Chronic HBV Infection

**FIGURE 3:**
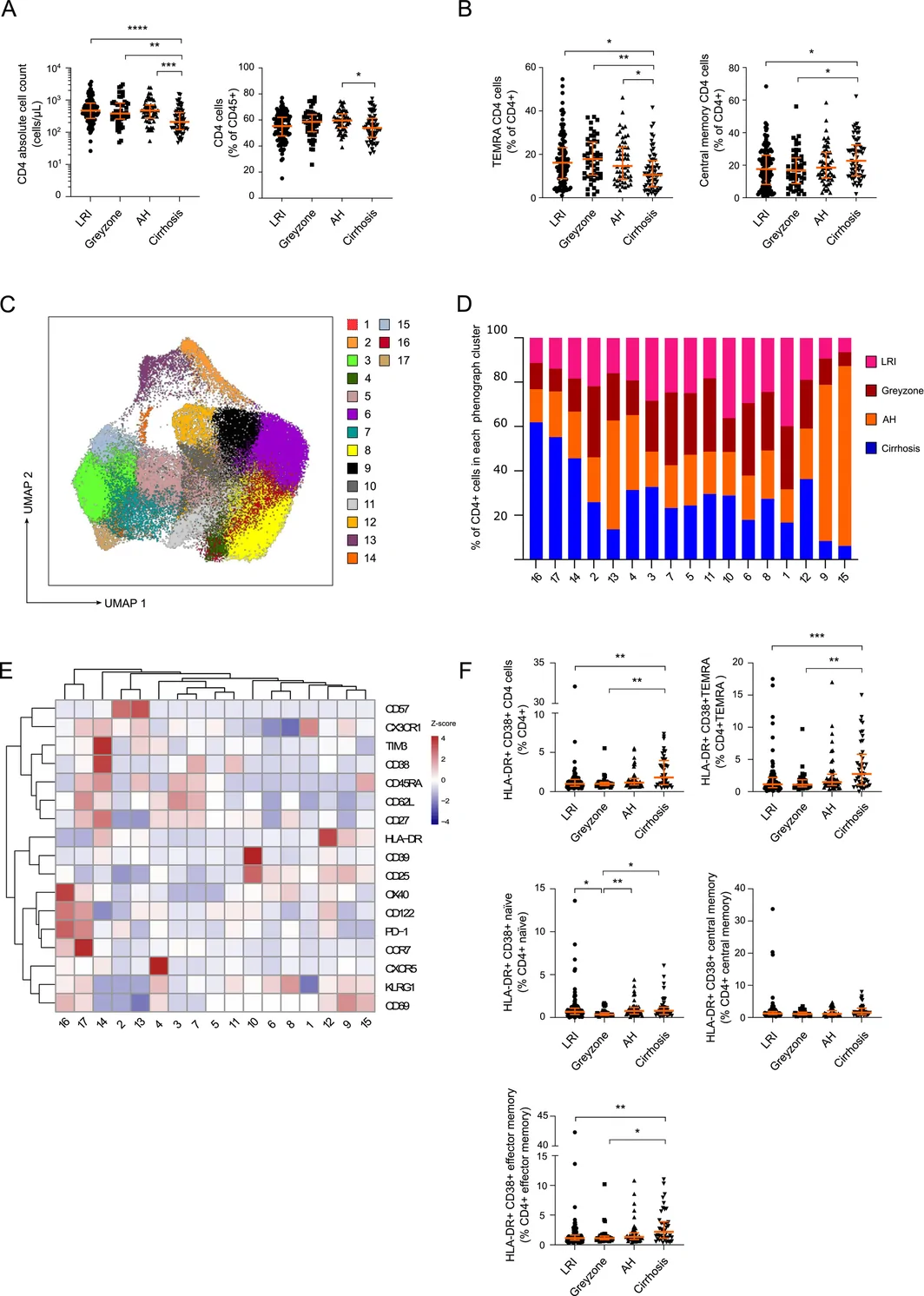
HLA‐DR^+^CD38^+^ CD4^+^ T cells are enriched in cirrhosis. (A) CD4 T cell absolute cell count (cells/μL) (left), frequency of CD4^+^ T cells (right). (B) Frequencies of terminal TEMRA CD4 T cells (CCR7^−^ CD45RA^+^) (left). Central memory CD4 T cells (CCR7^+^ CD45RA^−^) (right). Each dot represents one donor (*n* = 118 low‐replicative infection, *n* = 51 “greyzone”, *n* = 55 active hepatitis *n* = 63 cirrhosis). Line represents the median; error bars represent interquartile range. (C) UMAP of CD4 cells. 5000 cells from each of 4 patients from each clinical group (*n* = 16) were concatenated. Phenograph analysis overlaid on the UMAP showing 17 clusters in the CD4^+^ T cell compartment. (D) Stacked bar graph showing CD4^+^ T cell Phenograph clusters as % from each clinical group in the cluster. (E) Heatmap analysis displaying each individual CD4^+^ T cell Phenograph cluster (*x*‐axis) with median fluorescence intensity *z*‐score for markers used in panel 2 (*y*‐axis). Hierarchical clustering was done using flowcore (v2.20.0), pheatmap (v1.0.13), tidyverse (v.2.0.0) and dplyr (v1.1.4) packages in R studio. (F) HLA‐DR^+^ CD38^+^ CD4^+^ T cells percentage of total CD4^+^ T cell population (top left). HLA‐DR^+^ CD38^+^ TEMRA CD4^+^ T cells percentage of total TEMRA CD4^+^ T cell population (top right). HLA‐DR^+^ CD38^+^ naïve CD4^+^ T cells percentage of total naïve CD4^+^ T cell population (middle left). HLA‐DR^+^ CD38^+^ central memory CD4^+^ T cells percentage of total central memory CD4^+^ T cell population (middle right). HLA‐DR^+^ CD38^+^ effector memory CD4^+^ T cells percentage of total effector memory CD4^+^ T cell population (bottom left). Each dot represents one donor (*n* = 100 low‐replicative infection, *n* = 39 “greyzone”, *n* = 47 active hepatitis *n* = 43 cirrhosis). Line represents the median; error bars represent interquartile range. Statistical differences were assessed using Kruskal–Wallis test with post‐analysis Dunn's test *p < 0.05; **p < 0.01; ***p < 0.001; and ****p < 0.0001. Only significant differences are displayed. TEMRA, terminally differentiated effector memory re‐expressing. AH, acute hepatitis; LRI, low‐replicative infection; UMAP, uniform manifold approximation and projection.

CD4^+^ T cells are not frequently studied in HBV infection; however, antigen‐specific CD4^+^ T cells have been described to be reduced in chronic HBV infection and express high levels of the exhaustion marker PD‐1 [29]. In this study, CD4^+^ T cell populations were defined as CD3^+^TCRVα7.2^−^ CD161^−^CD8^−^CD4^+^. Absolute cell counts of CD4^+^ T cells were reduced in cirrhosis in comparison to all other clinical groups (208.9 vs. 470.4 cells/μL low‐replicative infection, 386 cells/μL “greyzone” and 473 cells/μL active hepatitis) (Figure 3A and Table S4). In addition, there was an observed shift in CD4^+^ T cell subgroups with a decreased frequency of terminal effector memory (TEMRA) CD4^+^ T cells (CCR7^−^CD45RA^+^) in cirrhosis compared to low‐replicative infection, “greyzone” and active hepatitis (median frequency 10.5% vs. 16.1%, 17.8% and 14.6% respectively) and a higher frequency of central memory CD4^+^ T cells (CCR7^+^CD45RA^−^) compared to low‐replicative infection and “greyzone” (median frequency 22.7% vs. 17.6% and 16.8%) (Figure 3B). CD4^+^ regulatory T cells (Tregs) (CD127^−^ CD25^+^) have been reported to fluctuate in HBV depending on disease stage, with increasing Tregs in chronic HBV and a subsequent decrease as patients enter the final stages of disease [30, 31]. In this cohort, Treg frequency was not altered significantly while the Treg absolute count revealed a significant reduction in the cirrhosis group compared to low‐replicative infection and active hepatitis (3.3 vs. 7.9 cells/μL and 6.1 cells/μL respectively) (Figure S5A).

Unsupervised clustering revealed 17 clusters in CD4^+^ T cells (after Treg exclusion) (Figures 3C–E and S6A). CD4^+^ T cells from cirrhosis predominated cluster 17, which showed expression of CCR7 and CD45RA together with CD27 and CD62L, indicating a naïve CD4^+^ T cell phenotype. However, the expression of CD122 within cluster 17 suggests a stem cell‐like memory T cell subtype [32]. Cluster 16 was highly represented in cirrhosis and displayed expression of OX40, PD‐1, and CD122, suggestive of an effector cluster with simultaneous signs of activation and exhaustion. Lastly, cluster 14 was prominent in cirrhosis and notably displayed high CD45RA and low CCR7 expression, suggestive of CD4^+^ TEMRA cells. Additionally, cluster 14 displayed expression of TIM3, suggestive of exhaustion but notably with higher expression of both CD38 and HLA‐DR. Looking at the overall frequencies of HLA‐DR^+^CD38^+^ CD4^+^ T cells, there was a significant increase of these double positive cells in cirrhosis compared with other clinical groups (median frequency 1.79% vs. 0.99% low‐replicative infection and 0.99% “greyzone”) and this trend was exaggerated in the CD4^+^ TEMRA cells (Figure 3F).

### 3.6 CD38 ^+^ HLA‐DR ^+^ CD8 ^+^ T Cells Are Increased in HBV‐Associated Cirrhosis

**FIGURE 4:**
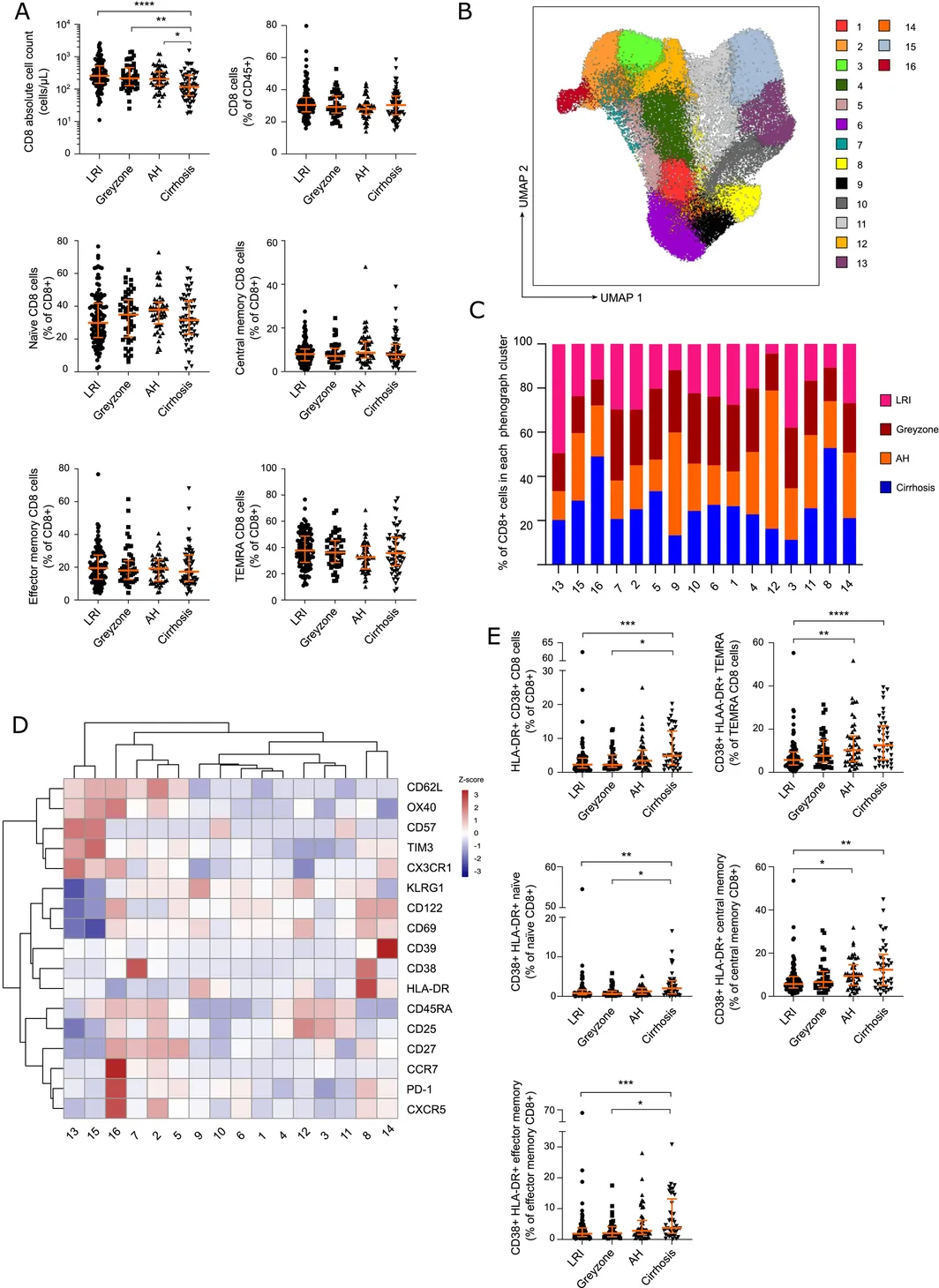
CD8^+^ T cell counts are reduced in cirrhosis and display expression of HLA‐DR and CD38. (A) CD8^+^ T cell absolute cell count (cells/μL) (top left), CD8^+^ T cells percentage of all lymphocytes (CD45) (top right). Naïve CD8 T cells (CCR7^+^ CD45RA^+^) percentage of all CD8^+^ T cells (middle left). Central memory CD8^+^ T cells (CCR7^+^ CD45RA^−^) percentage of all CD8^+^ T cells (middle right). Effector memory CD8 T cells (CCR7^−^ CD45RA^−^) percentage of all CD8^+^ T cells (bottom left). Terminal effector memory cells (TEMRA) CD8^+^ T cells (CCR^−^ CD45RA^+^) percentage of all CD8^+^ T cells (bottom right). Each dot represents one donor (*n* = 118 low‐replicative infection, *n* = 51 “greyzone”, *n* = 55 active hepatitis *n* = 63 cirrhosis). Line represents the median; error bars represent interquartile range. (B) UMAP with 5000 CD8^+^ cells from each of 4 patients from each clinical group (*n* = 16) were concatenated. Phenograph analysis showing 16 clusters in the CD8 T cell compartment. (C) Stacked bar graph showing CD8^+^ T cell Phenograph clusters as % from each clinical group in the cluster. (D) Heatmap analysis displaying each individual CD8^+^ T cell Phenograph cluster (*x*‐axis) with MFI *z*‐score for markers used in panel 2 (*y*‐axis). Hierarchical clustering was done using flowcore (v2.20.0), pheatmap (v1.0.13), tidyverse (v.2.0.0) and dplyr (v1.1.4) packages in R studio. (E) HLA‐DR^+^CD38^+^ CD8^+^ T cells percentage of total CD8^+^ T cell population (top left). HLA‐DR^+^CD38^+^ TEMRA CD8^+^ T cells percentage of TEMRA CD8^+^ T cell population (top right). HLA‐DR^+^CD38^+^ naïve CD8^+^ T cells percentage of naïve CD8^+^ T cell population (middle left). HLA‐DR^+^CD38^+^ central memory CD8^+^ T cells percentage of central memory CD8^+^ T cell population (middle right). HLA‐DR^+^CD38^+^ effector memory CD8^+^ T cells percentage of effector memory CD8^+^ T cell population (bottom left). Each dot represents one donor (*n* = 100 low‐replicative infection, *n* = 39 “greyzone”, *n* = 47 active hepatitis *n* = 43 cirrhosis). Line represents the median; error bars represent interquartile range. Statistical differences were assessed using Kruskal–Wallis test with post hoc Dunn's test *p < 0.05; **p < 0.01; ***p < 0.001; and ****p < 0.0001. Only significant differences are displayed. AH, acute hepatitis; LRI, low‐replicative infection; MFI, median fluorescence intensity; TEMRA, terminally differentiated effector memory re‐expressing; UMAP, uniform manifold approximation and projection.

Lastly, CD8^+^ T cells (CD3^+^TCRα7.2^−^CD161^−^CD8^+^) were analysed. Fewer absolute CD8^+^ T cell counts were observed in cirrhosis compared with all other clinical groups (118.3 vs. 255.3 cells/μL low‐replicative infection, 215.2 cells/μL “greyzone” and 205.7 cells/μL active hepatitis), while overall frequencies were comparable between the distinct groups (Figure 4A and Table S4). Assessment of the CD8^+^ T cell population revealed no differences in the frequency of effector/naïve/memory/terminal effector memory CD8^+^ T cells. MAIT cells were defined as CD3^+^ TCRα7.2^+^CD161^+^ and had a lower cell count in cirrhosis (1.67 vs. 3.26 cells/μL in low‐replicative infection and 3.46 cells/μL in active hepatitis) with no observed difference in the frequencies between the groups (Figure S5B).

Unsupervised clustering analysis identified 16 distinct CD8^+^ T cell clusters (Figures 4B–D and S6B). Cluster 8 was prominent within the cirrhosis group and displayed an increased expression of HLA‐DR and CD38. Taking the frequency of double positive HLA‐DR^+^CD38^+^ CD8^+^ T cells in the whole cohort there was an increased frequency in cirrhosis compared to low‐replicative infection and “greyzone” (median frequency 4.92% vs. 2.28% and 2.22% respectively) (Figure 4E). This was exaggerated in cirrhosis when looking specifically at the TEMRA CD8^+^ T cells compared to low‐replicative infection (median frequency 12.6% vs. 5.7% in low‐replicative infection) (Figure 4E).

### 3.7 Frequencies of HLA‐DR ^+^ CD38 ^+^ NK Cells and T Cells Correlate With the Degree of Liver Cirrhosis

**FIGURE 5:**
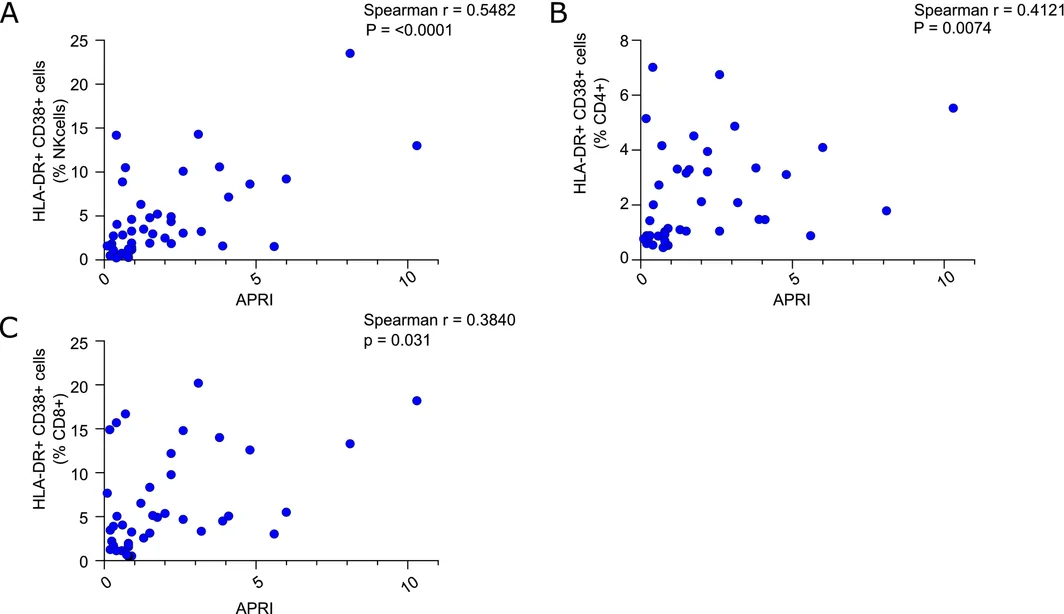
HLA‐DR^+^CD38^+^ immune cells correlate with cirrhosis severity. (A) HLA‐DR^+^CD38^+^ NK cells as a frequency of total NK cells are plotted on the *y*‐axis. AST to Platelet Ratio Index (APRI) score from cirrhosis patients calculated as [(AST (U/L)/ULN of AST) × 100]/platelet count (×10^9^/L). Each dot represents one donor. Correlation was assessed using Spearman's rank correlation (*R* = 0.5482, *p* < 0.0001). (B) HLA‐DR^+^CD38^+^ CD4^+^ T cells as a frequency of total CD4^+^ T cells are plotted on the *y*‐axis. APRI score from cirrhosis patients is plotted on the *x*‐axis. Each dot represents one donor. Correlation was assessed using Spearman's rank correlation (*R* = 0.4121, *p* = 0.0074) (C) HLA‐DR^+^CD38^+^ CD8^+^ T cells as a frequency of total CD8^+^ T cells are plotted on the *y*‐axis. APRI score from cirrhosis patients is plotted on the *x*‐axis. Each dot represents one donor. Correlation was assessed using Spearman's rank correlation (*R* = 0.3840, *p* = 0.031).

As CD38^+^ HLA‐DR^+^ NK cells, CD4^+^ and CD8^+^ T cells were observed as a common feature in cirrhosis, correlation analyses of these double positive populations with APRI score were performed (Figure 5). These identified positive correlations for the HLA‐DR^+^CD38^+^ NK cells (Spearman *r* = 0.5482, *p*‐value < 0.0001), HLA‐DR^+^CD38^+^ CD4^+^ T cells (Spearman *r* = 0.4121, *p*‐value = 0.0074), and HLA‐DR^+^CD38^+^ CD8^+^ T cells (Spearman *r* = 0.3840, *p*‐value = 0.031), suggesting that as disease severity increases, the double positive cell frequency increases.

## 4 Discussion

In a large cohort of Ethiopians with chronic HBV infection, patients with cirrhosis exhibited an immune phenotype clearly divergent from the other phases of HBV infection. Alterations of immune cell frequencies or markers in the “greyzone”, active hepatitis and low‐replicative hepatitis groups were less pronounced. Excepting an increased expression of CD38 on T cells in the active hepatitis group compared with the low‐replicative infection group (data not shown), we did not observe statistically significant differences between the three other groups. This may be due in part to the strong alterations of immune cell composition observed in cirrhosis. Across both the innate and adaptive immune cells, there was a significant reduction in the absolute cell counts in the cirrhosis group compared to other clinical groups, though a total lymphocyte count was not available to confirm. Lymphopenia is a common finding in patients with advanced cirrhosis [33], and previous gene expression profiles in patients with cirrhosis of varying aetiologies have described similar immune cell depletion [34].

In contrast to other immune cell subsets, monocytes are typically increased in cirrhosis [35], and the same observation was made in this cohort. Similarly, with monocyte differentiation, cirrhosis patients are reported to have higher percentages of intermediate monocytes and lower non‐classical monocytes when compared with healthy controls [35, 36], which we also observed. Furthermore, HLA‐DR expression on monocytes has been shown to decrease in decompensated liver disease [37]. In the present study, around half of the patients within the cirrhosis group had signs of decompensation; however, a further separation into decompensated and compensated cirrhosis was insufficiently robust for statistical testing. Therefore, the cirrhosis group included both compensated and decompensated individuals, and we did not observe any difference in HLA‐DR expression in cirrhosis compared with other clinical groups.

Overall, there was a shift from differentiated cells to a naïve immune cell phenotype within HBV‐associated cirrhosis. Previous studies have described a shift of B cells in liver cirrhosis from mature CD27^+^ memory B cells to a naïve CD27^−^ phenotype [38, 39]. CD4^+^ T cell abnormalities are less well‐described, although CD4^+^ TEMRA cells have recently been found to increase over time in alcohol‐related cirrhosis [40]. NK cells can be subdivided into two subsets based on their CD56 expression. CD56^dim^ cells predominate in blood and are more mature with higher cytotoxic capabilities, whilst CD56^bright^ cells are more abundant in tissue, are less differentiated, and are greater cytokine producers [41, 42]. A shift from CD56^dim^ to CD56^bright^ cells has been described previously in both acute and chronic HBV, suggestive of a more naïve phenotype in HBV infection [28, 43]. However, CD8^+^ T cells did not display the same shift in differentiation, a phenomenon which has also been previously described [44]. Excluding the stability seen in CD8^+^ T cells, the shift to a naïve phenotype, was not just an isolated event, but more of a collective effect, indicating changes in multiple immune cell subset distributions in patients with advanced disease.

Cirrhosis‐associated immune dysfunction (CAID) is a term used to describe the immunological abnormalities observed in patients with advanced liver disease of all aetiologies and describes the immune deficiency and systemic inflammation observed in cirrhosis [45, 46]. This study confirms these observations and strengthens the argument that the immunophenotype of cirrhosis is independent of the underlying aetiology. Once a patient progresses to end stage liver disease there are several factors that contribute to systemic inflammation and activation of immune cells such as gut bacterial translocation, loss of tolerance in the liver with increased pro‐inflammatory cytokines and hepatocyte death [45]. One key feature that helps define CAID is the development of CD38^+^HLA‐DR^+^ CD8^+^ T cells [44, 45]. While CD38 and HLA‐DR are generally markers of activation, CD8^+^ T cells that displayed high expression of CD38 and HLA‐DR have also been shown to express inhibitory markers including PD‐1, CTLA‐4 and TIM‐3 [44]. This was confirmed in the clustering analysis with increased PD‐1 expression in the double positive cells. This ‘exhausted’ double positive population was not exclusively seen in CD8^+^ T cells but also in CD4^+^ T cells which expressed TIM‐3 in addition to CD38 and HLA‐DR. In chronic HIV infection, a positive correlation of TIM‐3 and CD38 expression with viral load has been previously described, marking a dysfunctional T cell population [47]. On a transcriptional level, HLA‐DR^+^CD38^+^ CD8^+^ T cells express high levels of granzyme B and perforin but mainly contribute to liver damage in cirrhosis instead of viral clearance [48]. Furthermore, a decrease in CD38^+^ and HLA‐DR^+^CD38^+^ CD8^+^ T cells has been described to accompany viral load and HBsAg decline respectively in chronic HBV patients undergoing successful antiviral treatment [49, 50]. NK cells in cirrhosis patients displayed increased expression of both CD38 and HLA‐DR, as well as the activating receptor NKp46 and NKG2D. NKp46 and NKG2D have been previously reported to be elevated in chronic HBV infection [51, 52], and also implicated in the development of liver inflammation [51]. Similarly, previous studies have shown an increasing frequency of HLA‐DR^+^CD38^+^ NK cells as ALT increases [43]. In the present study the HLA‐DR^+^CD38^+^ cell populations of NK cells particularly, but also CD4^+^ T cells and CD8^+^ T cells, correlated with APRI score, used to estimate the degree of fibrosis/cirrhosis, indicating that the occurrence of these cells may contribute to liver damage.

This study had some limitations. First, although it provides valuable insights into the phenotype of African patients with cirrhosis, the cells were fixed which prevents any functional analysis. Therefore, it cannot be determined that the shift from effector to naïve phenotype correlates with a decrease in functional activity. Second, this was a cross‐sectional study, and therefore no conclusions can be drawn regarding the dynamics of the observed immune cell changes. In addition, no follow‐up visits were recorded, which could have resulted in the inclusion of a small number of acute HBV patients. However, most HBV infections in the Ethiopian region are acquired in childhood with only 1% of patients in a similar cohort having an acute HBV infection [53]. Third, although newer whole blood cryopreservation methods are comparable with standard PBMC freezing methods for general immune phenotyping, some cell markers have been less reliably detected [54], something which we observed, for example with PD‐1. Despite working with different clones and fluorochromes we did not detect a significant difference in PD‐1 expression across clinical stages of disease, as has been described previously, which could explain why we did not observe more subtle differences between the low replicative infection, “greyzone” and active hepatitis groups [55]. We used a small volume of blood which in some ways was advantageous as samples had to be shipped and stored, and this negates the need for a large blood draw as is the case for PBMC collection. We were still able to identify less frequent immune cell subsets, including dendritic cells and MAIT cells; however, due to the small blood volume analysed, significant differences in low frequency cell types might be underestimated. Lastly, this analysis used peripheral blood cells as a proxy for the immunological changes in liver disease, therefore likely not capturing all immunological changes associated with chronic HBV infection. ScRNAseq studies show clear differences in immune subsets, particularly myeloid cells, when liver samples are compared with peripheral blood [56, 57, 58]. Ultimately to understand the full picture of immune dysfunction in cirrhosis and what is driving pathogenesis, liver samples are required alongside detailed functional analysis in a longitudinal cohort.

The design of this study had several key strengths which support the robustness of the findings. First, the size of the cohort, including 287 untreated HBV‐infected individuals, ensured a large sample size across all clinical phases. Second, the clinical categories were clearly defined, and patients that did not fit into either category were removed to avoid dilution. Using a simple cryopreservation method allowed for sampling of a large Ethiopian cohort and paves the way for improved representation and personalized treatment for African populations with HBV. Furthermore, by using high‐dimensional flow cytometry it was possible to incorporate phenotyping across all immune cells in peripheral blood and describe collectively the immune dysfunction observed in cirrhosis.

In conclusion, in this cohort of untreated, chronically infected HBV patients in Ethiopia, cirrhosis displayed a complex immune signature characterized by simultaneous activation and exhaustion. Furthermore, the cirrhosis‐associated immune dysfunction phenotype was marked by the emergence of HLA‐DR^+^CD38^+^ NK cell and T cell populations, indicating that the imprint of cirrhosis on immune cell populations is conserved across ethnicities and aetiologies of liver cirrhosis.

## Author Contributions

**Julia MacLeod:** conceptualization, writing – original draft, formal analysis, data curation, methodology, investigation, visualization, validation. **Fikadu G. Gudissa:** writing – review and editing, resources. **Asiya Jeylan:** writing – review and editing, resources. **Nega Berhe:** writing – review and editing, resources. **Hailemichael Desalegn:** writing – review and editing, resources. **Lasse Rossvoll:** resources, writing – review and editing, data curation. **Asgeir Johannessen:** data curation, resources, funding acquisition, writing – review and editing, conceptualization, writing – original draft, project administration, supervision. **Niklas K. Björkström:** conceptualization, funding acquisition, writing – review and editing, resources, project administration, methodology, supervision. **Dag Henrik Reikvam:** conceptualization, funding acquisition, writing – review and editing, data curation, writing – original draft, project administration, supervision. **Annika Niehrs:** conceptualization, investigation, writing – review and editing, writing – original draft, methodology, validation, supervision, resources, project administration, formal analysis, data curation.

## Funding

This work was funded by the Research Council of Norway (grant ID 336567) with an additional contribution from the Nordic Society of Clinical Microbiology and Infectious Diseases. The Ethiopian HBV cohort was funded by the South‐Eastern Norway Regional Health Authority (Helse Sør‐Øst, https://www.helse‐sorost.no, grant number 2021064) and the John C Martin Foundation (https://thejcmfoundation.org/, grant number 2021‐G036). In addition to the European Research Council (ERC) under the European Union's Horizon 2020 research and innovation program (grant agreement No. 948692), the Swedish Research Council (grant number 2025‐06655), the Swedish Cancer Society, the Swedish Foundation for Strategic Research, the Knut and Alice Wallenberg Foundation, the Center for Innovative Medicine at Karolinska Institutet, Region Stockholm, the NovoNordisk Foundation, and Karolinska Institutet provided funding.

## Conflicts of Interest

The authors declare no conflicts of interest.

## Supporting information


**Figure S1:** Gating strategy. Gating strategy for identifying immune cell populations using FlowJo (v10.10.0). Leukocytes and counting beads were identified. Neutrophils were excluded due to poor CD45 staining on CD15^+^ cells and inefficiency to identify individual pooled CD45 populations. Individual patients were identified based on CD45 staining. Samples were subsequently gated based on lineage markers T cells, B cells, monocytes, dendritic cells, ILCs and NK cells and further gated into respective subpopulations. ILC, innate lymphoid cells; NK, natural killer.
**Figure S2:** Quantitative HBV s antigen is higher in “greyzone” group. qHBsAg (IU/mL) scatter plot in four clinical groups—low‐replicative infection, “greyzone”, active hepatitis and cirrhosis. Each dot represents one donor (*n* = 114 low‐replicative infection, *n* = 48 “greyzone”, *n* = 52 active hepatitis *n* = 59 cirrhosis). Line represents the median; error bars represent interquartile range. Statistical differences were assessed using Kruskal–Wallis test with post hoc Dunn's test *p < 0.05; **p < 0.01; and ***p < 0.001. LRI, low‐replicative infection; AH, active hepatitis; qHBsAg, quantitative Hepatitis B s Antigen.
**Figure S3:** NK cell heatmaps. (A) Concatenated CD56^+^ NK cells from four patients from each clinical group presented as a UMAP analysis with each colour representing the cells from that clinical group; pink—low‐replicative infection, red—“greyzone”, orange—active hepatitis and blue—cirrhosis (left). (B) Heatmap with each individual marker expression for CD56, CD16, CD57, NKG2A, CD38 and HLA‐DR in NK cells (right). Red circles highlighting clusters positive for CD38 and/or HLA‐DR prominent in the cirrhosis UMAP. NK, natural killer; UMAP, uniform manifold approximation and projection.
**Figure S4:** B cell maturation regresses with disease progression in chronic HBV. (A) B cell absolute count (cells/μL) (left). B cell frequency (%) of total lymphocytes (CD45^+^ cells) (right). (B) Switched memory B cells (CD27^+^ IgD^−^) frequency (%) of total clean B cells (left). Naïve B cells (CD27^−^ IgD^+^) frequency (%) of total clean B cells (right). Line represents the median; error bars represent interquartile range. Statistical differences were assessed using Kruskal–Wallis test with post hoc Dunn's test *p < 0.05; **p < 0.01; and ***p < 0.001. LRI, low‐replicative infection; AH, active hepatitis.
**Figure S5:** MAIT cell and Regulatory T cell counts are reduced in cirrhosis. (A) Regulatory CD4^+^ T cells (Treg) absolute count (cells/μL) (left). Treg cell frequency (%) of total lymphocytes (CD45^+^) cells) (right). (B) MAIT cell absolute count (cells/μL) (left). MAIT cell frequency (%) of total lymphocytes (CD45^+^ cells) (right). D45^+^ Line represents the median; error bars represent interquartile range. Statistical differences were assessed using Kruskal–Wallis test with post hoc Dunn's test *p < 0.05; **p < 0.01; and ***p < 0.001. LRI, low‐replicative infection; AH, active hepatitis; MAIT, mucosal‐associated invariant T cell; Treg, Regulatory T cell.
**Figure S6:** CD4 and CD8 T cell Heatmaps. (A) Concatenated CD4^+^ T cells from four patients from each clinical group presented as a UMAP analysis (left). Heatmap with each individual marker expression for CD45RA, CCR7, CD38 and HLA‐DR in CD4 T cells (right). Red circles highlighting clusters positive for CD38 and/or HLA‐DR prominent in the cirrhosis UMAP. (B) Concatenated CD8^+^ T cells from four patients from each clinical group presented as a UMAP analysis (left). Heatmap with each individual marker expression for CD45RA, CCR7, CD38 and HLA‐DR in CD8 T cells (right). Red circles highlighting clusters positive for CD38 and/or HLA‐DR prominent in the cirrhosis UMAP. UMAP, uniform manifold approximation and projection.
**Table S1:** Patient characteristics.
**Table S2:** List of antibodies with supplier, catalogue number and clone.
**Table S3:** Hierarchical gating strategy for all major immune cell subtypes.
**Table S4:** Median [interquartile range] absolute cell counts of major cell groups given as cells/μL for each clinical group.

## Data Availability

All primary data is available upon request from the corresponding authors.
