# Supplementary material for: A Distinct Immune Signature Characterizes Cirrhosis in Ethiopian Individuals With Chronic HBV Infection
Source: Aliment Pharmacol Ther. 2026 Jul 1;64(7):951–64. doi: 10.1111/apt.70816 (PMC13559501; doi:10.1111/apt.70816)
Supplement: Supplementary file 1 [file APT-64-951-s001.docx]

Supplementary data

**A distinct immune signature characterizes cirrhosis in Ethiopian Individuals with Chronic HBV Infection**

**Authors**: Julia MacLeod^1,2^, Fikadu G Gudissa^3^, Asiya Jeylan^3^, Nega Berhe^4,5^, Hailemichael Desalegn^4,6^, Lasse Rossvoll^1,4^, Asgeir Johannessen^1,4^_,_ Niklas K. Björkström^7^, Dag Henrik Reikvam^1,2*^, Annika Niehrs^7*^

^1^Institute of Clinical Medicine, University of Oslo, Oslo, Norway.

^2^Department of Infectious Diseases, Oslo University Hospital, Oslo, Norway.

^3^Adama Comprehensive Specialized Hospital Medical College, Adama, Ethiopia.

^4^Department of Infectious Diseases, Vestfold Hospital, Tønsberg, Norway.

^5^Aklilu Lemma Institute of Pathobiology, Addis Ababa University, Addis Ababa, Ethiopia.

^6^St. Paul’s Hospital Millenium Medical College, Addis Ababa, Ethiopia.

^7^Centre for Infectious Medicine, Department of Medicine Huddinge, Karolinska Institutet, Karolinska University Hospital, Stockholm, Sweden.

*Corresponding authors: Addresses: Institute of Clinical Medicine, University of Oslo, Oslo, Norway (Dag Henrik Reikvam) or Centre for Infectious Medicine, Department of Medicine Huddinge, Karolinska Institutet, Karolinska University Hospital, Stockholm, Sweden (Annika Niehrs); Telephone: +47 94858939 (Dag Henrik Reikvam) or +4673-5018261 (Annika Niehrs) E-Mail: [d.h.reikvam@medisin.uio.no](mailto:d.h.reikvam@medisin.uio.no) (Dag Henrik Reikvam) and [annika.niehrs@ki.se](mailto:annika.niehrs@ki.se) (Annika Niehrs)

**Table of contents**

Figure S1	p3

Figure S2	p5

Figure S3	p6

Figure S4	p7

Figure S5	p8

Figure S6	p9

Table S1	p10

Table S2	p19

Table S3	p22

Table S4	p25

**Supplementary Figure 1**

**Fig.S1. Gating strategy**

Gating strategy for identifying immune cell populations using FlowJo (v10.10.0). Leukocytes and counting beads were identified. Neutrophils were excluded due to poor CD45 staining on CD15+ cells and inefficiency to identify individual pooled CD45 populations. Individual patients were identified based on CD45 staining. Samples were subsequently gated based on lineage markers T cells, B cells, monocytes, dendritic cells, ILCs and NK cells and further gated into respective subpopulations. ILC, innate lymphoid cells; NK, natural killer.

**Supplementary Figure 2**

**Fig. S2. Quantitative HBV s antigen is higher in “Greyzone” group**

qHBsAg (IU/mL) scatter plot in four clinical groups – low-replicative infection, “greyzone”, active hepatitis and cirrhosis. Each dot represents one donor (n= 114 low-replicative infection, n=48 “greyzone”, n=52 active hepatitis n=59 cirrhosis). Line represents the median; error bars represent interquartile range. Statistical differences were assessed using Kruskal-Wallis test with *post hoc* Dunn’s test ∗*p <*0.05; ∗∗*p <*0.01; and ∗∗∗*p <*0.001. LRI, low-replicative infection; AH, active hepatitis; qHBsAg, quantitative Hepatitis B s Antigen

**Supplementary Figure 3**

**Fig. S3. NK cell Heatmaps**

(A) Concatenated CD56^+^ NK cells from four patients from each clinical group presented as a UMAP analysis with each colour representing the cells from that clinical group; pink – low replicative infection, red – “greyzone”, orange – active hepatitis and blue – cirrhosis (left). (B) Heatmap with each individual marker expression for CD56, CD16, CD57, NKG2A, CD38 and HLA-DR in NK cells (right). Red circles highlighting clusters positive for CD38 and/or HLA-DR prominent in the cirrhosis UMAP. NK, natural killer; UMAP, uniform manifold approximation and projection.

**Supplementary Figure 4**

**Fig. S4. B cell maturation regresses with disease progression in chronic HBV**

(A) B cell absolute count (cells/µL) (left). B cell frequency (%) of total lymphocytes (CD45+ cells) (right). (B) Switched memory B cells (CD27+ IgD-) frequency (%) of total clean B cells (left). Naïve B cells (CD27- IgD+) frequency (%) of total clean B cells (right). Line represents the median; error bars represent interquartile range. Statistical differences were assessed using Kruskal-Wallis test with *post hoc* Dunn’s test ∗*p <*0.05; ∗∗*p <*0.01; and ∗∗∗*p <*0.001. LRI, low-replicative infection; AH, active hepatitis.

**Supplementary Figure 5**

**Fig. S5. MAIT cell and Regulatory T cell counts are reduced in cirrhosis**

(A) Regulatory CD4 T cells (Treg) absolute count (cells/µL) (left). Treg cell frequency (%) of total lymphocytes (CD45+ cells) (right). (B) MAIT cell absolute count (cells/µL) (left). MAIT cell frequency (%) of total lymphocytes (CD45+ cells) (right) Line represents the median; error bars represent interquartile range. Statistical differences were assessed using Kruskal-Wallis test with *post hoc* Dunn’s test ∗*p <*0.05; ∗∗*p <*0.01; and ∗∗∗*p <*0.001. LRI, low-replicative infection; AH, active hepatitis; MAIT, mucosal-associated invariant T cell; Treg, Regulatory T cell.

**Supplementary Figure 6**

**Fig. S6. CD4 and CD8 T cell Heatmaps**

(A) Concatenated CD4^+^ T cells from four patients from each clinical group presented as a UMAP analysis (left). Heatmap with each individual marker expression for CD45RA, CCR7, CD38 and HLA-DR in CD4 T cells (right). Red circles highlighting clusters positive for CD38 and/or HLA-DR prominent in the cirrhosis UMAP. (B) Concatenated CD8^+^ T cells from four patients from each clinical group presented as a UMAP analysis (left). Heatmap with each individual marker expression for CD45RA, CCR7, CD38 and HLA-DR in CD8 T cells (right). Red circles highlighting clusters positive for CD38 and/or HLA-DR prominent in the cirrhosis UMAP. UMAP, uniform manifold approximation and projection.

**Table S1 Patient characteristics**

|  | **Sex:** | **Age** | **Platelets (10^9/L):** | **ALT (IU/L):** | **AST (IU/L):** | **APRI:** | **HBV DNA (IU/mL):** | **Liver stiffness (kPa):** | **Ultrasound** | **Group** | **anti-HDV** |
| --- | --- | --- | --- | --- | --- | --- | --- | --- | --- | --- | --- |
| 1 | Male | 35 | 283 | 19 | 29 | 0,26 | 5920 | 5,9 |  | “Greyzone” |  |
| 2 | Female | 33 | 247 | 29 | 20 | 0,20 | 0 |  |  | Low-replicative infection |  |
| 3 | Male | 52 | 53 | 33 | 37 | 1,7 | 51100000 |  |  | Cirrhosis |  |
| 4 | Female | 20 | 316 | 9 | 18 | 0,14 | 1260 |  | Normal | Low-replicative infection |  |
| 5 | Female | 66 | 436 | 14 | 23 | 0,13 | 42 |  |  | Low-replicative infection |  |
| 6 | Male | 35 | 84 | 37 | 98 | 2,92 | 163000 |  | Cirrhosis | Cirrhosis |  |
| 7 | Female | 27 | 307 | 23 | 26 | 0,21 | 3350 | 5,7 |  | “Greyzone” |  |
| 8 | Female | 35 | 230 | 14 | 20 | 0,22 | 461 |  |  | Low-replicative infection |  |
| 9 | Male | 40 | 160 | 32 | 39 | 0,61 | 20300 |  | Cirrhosis, PHT, splenomegaly | Cirrhosis |  |
| 10 | Male | 19 | 343 | 22 | 29 | 0,21 | 7,06E+08 |  |  | “Greyzone” |  |
| 11 | Male | 37 | 186 | 35 | 65 | 0,87 | 35 | 18,4 | Cirrhosis | Cirrhosis | Positive |
| 12 | Female | 20 | 417 | 18 | 26 | 0,16 | 1740 |  |  | Low-replicative infection |  |
| 13 | Male | 24 | 231 | 87 | 50 | 0,54 | 20100000 | 6,1 |  | Cirrhosis - decomp |  |
| 14 | Male | 30 | 251 | 19 | 25 | 0,25 | 1540 | 6,8 |  | Low-replicative infection |  |
| 15 | Female | 25 | 255 | 32 | 25 | 0,25 | 26 |  | Normal | Low-replicative infection |  |
| 16 | Male | 42 | 61 | 38 | 54 | 2,21 | 36500 | 5,1 |  | Cirrhosis - decomp |  |
| 17 | Male | 25 | 112 | 44 | 42 | 0,94 | 140000 | 6,6 | Cirrhosis, ascites, PHT, splenomegaly | Cirrhosis-decomp |  |
| 18 | Male | 32 | 172 | 61 | 52 | 0,76 | 154000 |  |  | Active hepatitis |  |
| 19 | Male | 25 | 301 | 34 | 32 | 0,27 | 0 |  |  | Low-replicative infection |  |
| 20 | Male | 45 | 138 | 25 | 50 | 0,91 | 79600 | 10,9 | Cirrhosis | Cirrhosis |  |
| 21 | Male | 29 | 184 | 91 | 56 | 0,76 | 13300 |  | Normal | Active hepatitis |  |
| 22 | Female | 23 | 199 | 18 | 19 | 0,24 | 950000 | 5,2 |  | “Greyzone” |  |
| 23 | Female | 27 | 292 | 19 | 17 | 0,15 | 0 |  |  | Low-replicative infection |  |
| 24 | Female | 36 | 142 | 18 | 12 | 0,21 | 0 |  |  | Low-replicative infection |  |
| 25 | Female | 20 | 574 | 22 | 49 | 0,21 | 59 |  | Normal | Cirrhosis - decomp |  |
| 26 | Male | 70 | 341 | 10 | 11 | 0,08 | 230 | 7,8 | Normal | Low-replicative infection |  |
| 27 | Female | 57 | 75 | 31 | 39 | 1,30 | 1,46E+08 | 35,3 | Cirrhosis | Cirrhosis |  |
| 28 | Male | 37 | 188 | 22 | 15 | 0,20 | 9290 |  |  | “Greyzone” |  |
| 29 | Female | 30 | 246 | 9 | 20 | 0,20 | 325 |  |  | Low-replicative infection |  |
| 30 | Male | 38 | 305 | 32 | 31 | 0,25 | 32 | 4 | Normal | Low-replicative infection |  |
| 31 | Male | 23 | 271 | 15 | 13 | 0,12 | 15 |  | Normal | Low-replicative infection |  |
| 32 | Male | 48 | 101 | 77 | 82 | 2,03 | 5660000 |  | Cirrhosis, PHT | Cirrhosis - decomp |  |
| 33 | Male | 25 | 270 | 19 | 25 | 0,23 | 621 |  | Normal | Low-replicative infection |  |
| 34 | Male | 21 | 258 | 74 | 59 | 0,57 | 2E+08 |  | Normal | Active hepatitis |  |
| 35 | Male | 42 | 305 | 30 | 33 | 0,27 | 1210 |  |  | Low-replicative infection |  |
| 36 | Male | 25 | 217 | 47 | 48 | 0,55 | 4060 |  | Normal | Active hepatitis |  |
| 37 | Male | 42 | 516 | 22 | 19 | 0,09 | 5010 |  | Normal | “Greyzone” |  |
| 38 | Female | 25 | 265 | 24 | 29 | 0,27 | 5880 | 5 |  | “Greyzone” |  |
| 39 | Male | 32 | 281 | 29 | 24 | 0,21 | 8280 |  |  | “Greyzone” |  |
| 40 | Female | 23 | 236 | 20 | 24 | 0,25 | 183 | 4,4 |  | Low-replicative infection |  |
| 41 | Female | 36 | 205 | 13 | 16 | 0,20 | 837 | 4,8 |  | Low-replicative infection |  |
| 42 | Female | 25 | 261 | 79 | 55 | 0,53 | 2270 |  | Normal | Active hepatitis |  |
| 43 | Female | 22 | 281 | 15 | 13 | 0,12 | 27 |  |  | Low-replicative infection |  |
| 44 | Female | 23 | 266 | 15 | 18 | 0,17 | 268 |  |  | Low-replicative infection |  |
| 45 | Female | 37 | 286 | 21 | 23 | 0,20 | 2900 | 5 |  | “Greyzone” |  |
| 46 | Female | 27 | 300 | 13 | 12 | 0,10 | 1590 |  | Normal | Low-replicative infection |  |
| 47 | Male | 49 | 158 | 12 | 14 | 0,22 | 199 |  |  | Low-replicative infection | Positive |
| 48 | Male | 30 | 270 | 19 | 20 | 0,19 | 1280 |  |  | Low-replicative infection |  |
| 49 | Female | 19 | 337 | 20 | 16 | 0,12 | 3900 | 5,6 |  | “Greyzone” |  |
| 50 | Male | 29 | 202 | 113 | 131 | 1,62 | 6050000 | 20 | Cirrhosis | Cirrhosis |  |
| 51 | Male | 40 | 273 | 35 | 30 | 0,27 | 3470 | 4,6 |  | “Greyzone” |  |
| 52 | Female | 25 | 260 | 21 | 21 | 0,20 | 3340 |  |  | “Greyzone” |  |
| 53 | Female | 42 | 334 | 17 | 17 | 0,13 | 45 | 3 | Normal | Low-replicative infection |  |
| 54 | Female | 22 | 247 | 26 | 24 | 0,24 | 2650 |  | Normal | “Greyzone” |  |
| 55 | Female | 19 | 308 | 13 | 24 | 0,19 | 1680 |  |  | Low-replicative infection |  |
| 56 | Male | 35 | 241 | 25 | 22 | 0,23 | 978 |  |  | Low-replicative infection |  |
| 57 | Male | 54 | 251 | 30 | 21 | 0,21 | 884 |  |  | Low-replicative infection |  |
| 58 | Female | 28 | 225 | 17 | 18 | 0,20 | 801 |  |  | Low-replicative infection |  |
| 59 | Female | 32 | 240 | 41 | 39 | 0,41 | 632000 | 3,3 | Normal | Active hepatitis |  |
| 60 | Female | 30 | 274 | 17 | 17 | 0,16 | 36 | 3,5 |  | Low-replicative infection |  |
| 61 | Female | 30 | 248 | 17 | 24 | 0,24 | 1080 |  |  | Low-replicative infection |  |
| 62 | Female | 40 | 270 | 30 | 29 | 0,27 | 948 |  | Normal | Low-replicative infection |  |
| 63 | Male | 26 | 284 | 40 | 28 | 0,25 | 2550 | 5,1 | Cirrhosis | Cirrhosis |  |
| 64 | Male | 43 | 402 | 9 | 18 | 0,11 | 525 | 4,8 |  | Low-replicative infection |  |
| 65 | Female | 35 | 244 | 18 | 22 | 0,23 | 356 | 4,7 | Normal | Low-replicative infection |  |
| 66 | Male | 31 | 353 | 32 | 25 | 0,18 | 524 | 5,6 | Cirrhosis | Cirrhosis |  |
| 67 | Female | 32 | 327 | 34 | 18 | 0,14 | 41300 |  |  | “Greyzone” |  |
| 68 | Female | 25 | 402 | 24 | 20 | 0,12 | 621 |  | Unspecific liver pathology | Cirrhosis-decomp |  |
| 69 | Male | 23 | 167 | 70 | 53 | 0,79 | 1300000 |  |  | Active hepatitis |  |
| 70 | Female | 28 | 739 | 22 | 22 | 0,07 | 554 |  |  | Low-replicative infection |  |
| 71 | Female | 20 | 394 | 38 | 13 | 0,08 | 22 | 4 | Normal | Low-replicative infection |  |
| 72 | Female | 22 | 275 | 14 | 17 | 0,15 | 585 |  | Normal | Low-replicative infection |  |
| 73 | Male | 50 | 136 | 57 | 43 | 0,79 | 128000 |  | Unspecific liver pathology | Active hepatitis |  |
| 74 | Male | 32 | 223 | 26 | 21 | 0,24 | 844 |  |  | Low-replicative infection |  |
| 75 | Male | 25 | 346 | 19 | 32 | 0,23 | 81 |  | Normal | Low-replicative infection |  |
| 76 | Female | 22 | 418 | 22 | 32 | 0,19 | 230 |  |  | Low-replicative infection |  |
| 77 | Female | 25 | 78 | 54 | 79 | 2,53 | 5920000 |  | Cirrhosis, ascites, PHT splenomegaly | Cirrhosis-decomp |  |
| 78 | Male | 34 | 168 | 17 | 13 | 0,19 | 24500 | 4 | Normal | “Greyzone” |  |
| 79 | Male | 25 | 106 | 30 | 38 | 0,90 | 1710000 |  | Cirrhosis, PTH, splenomegaly | Cirrhosis |  |
| 80 | Female | 28 | 264 | 15 | 17 | 0,16 | 41 | 4,3 | Normal | Low-replicative infection |  |
| 81 | Male | 25 | 206 | 23 | 8 | 0,10 | 3250 | 5,6 |  | “Greyzone” |  |
| 82 | Male | 62 | 206 | 46 | 52 | 0,63 | 117 |  | Cirrhosis, ascites | Cirrhosis - decomp |  |
| 83 | Female | 22 | 237 | 25 | 9 | 0,09 | 2060 | 4,3 | Normal | “Greyzone” |  |
| 84 | Male | 30 | 220 | 120 | 660 | 7,50 | 12800 |  |  | Active hepatitis |  |
| 85 | Male | 34 | 270 | 49 | 6 | 0,06 | 1210000 | 10,3 |  | Active hepatitis |  |
| 86 | Male | 24 | 134 | 77 | 71 | 1,32 | 60100 | 11,7 |  | Active hepatitis |  |
| 87 | Male | 32 | 154 | 570 | 411 | 6,67 | 2,07E+08 |  | Normal | Active hepatitis |  |
| 88 | Male | 33 | 314 | 27 | 31 | 0,25 | 36500 |  |  | “Greyzone” |  |
| 89 | Female | 33 | 240 | 13 | 23 | 0,24 | 227 |  |  | Low-replicative infection |  |
| 90 | Female | 30 | 271 | 10 | 19 | 0,18 | 10 |  |  | Low-replicative infection |  |
| 91 | Male | 25 | 267 | 25 | 21 | 0,20 | 18 | 5,6 |  | Low-replicative infection |  |
| 92 | Female | 22 | 298 | 27 | 30 | 0,25 | 10 |  |  | Low-replicative infection |  |
| 93 | Male | 32 | 179 | 52 | 54 | 0,75 | 45800 |  | Normal | Active hepatitis |  |
| 94 | Female | 18 | 244 | 88 | 35 | 0,36 | 40600000 | 6,2 | Unspecific liver pathology | Active hepatitis |  |
| 95 | Female | 28 | 268 | 13 | 22 | 0,21 | 20 |  |  | Low-replicative infection |  |
| 96 | Female | 43 | 249 | 15 | 20 | 0,20 | 950 | 3,7 |  | Low-replicative infection |  |
| 97 | Male | 26 | 160 | 44 | 27 | 0,42 | 5210 |  | Normal | Active hepatitis |  |
| 98 | Female | 50 | 270 | 11 | 20 | 0,19 | 17 |  |  | Low-replicative infection |  |
| 99 | Female | 24 | 317 | 11 | 16 | 0,13 | 80 |  | Normal | Low-replicative infection |  |
| 100 | Female | 24 | 264 | 12 | 18 | 0,17 | 143 |  | Normal | Low-replicative infection |  |
| 101 | Male | 29 | 144 | 49 | 46 | 0,80 | 1190000 |  |  | Active hepatitis |  |
| 102 | Male | 29 | 358 | 18 | 40 | 0,28 | 1690 | 6,1 | Normal | Low-replicative infection |  |
| 103 | Male | 23 | 85 | 75 | 64 | 1,88 | 44200000 |  |  | Active hepatitis |  |
| 104 | Female | 39 | 66 | 23 | 32 | 1,21 | 329 |  |  | Cirrhosis - decomp |  |
| 105 | Male | 60 | 260 | 22 | 26 | 0,25 | 1050 | 6,1 |  | Low-replicative infection |  |
| 106 | Female | 37 | 215 | 41 | 46 | 0,53 | 14800000 |  | Unspecific liver pathology | Active hepatitis |  |
| 107 | Female | 20 | 378 | 10 | 17 | 0,11 | 125 |  |  | Low-replicative infection |  |
| 108 | Female | 29 | 263 | 19 | 26 | 0,25 | 7070 |  | Cirrhosis | Cirrhosis |  |
| 109 | Male | 57 | 275 | 60 | 44 | 0,40 | 440000 |  |  | Active hepatitis |  |
| 110 | Male | 32 | 42 | 55 | 80 | 4,76 | 292 |  |  | Cirrhosis - decomp |  |
| 111 | Male | 46 | 276 | 40 | 11 | 0,10 | 1350 | 5,3 | Normal | Low-replicative infection |  |
| 112 | Female | 28 | 136 | 76 | 50 | 0,92 | 8000000 |  | Unspecific liver pathology | Active hepatitis |  |
| 113 | Male | 42 | 233 | 18 | 21 | 0,23 | 0 | 5,8 |  | Low-replicative infection |  |
| 114 | Male | 21 | 54 | 32 | 80 | 3,70 | 40900 | 19,2 |  | Cirrhosis |  |
| 115 | Male | 23 | 266 | 11 | 12 | 0,11 | 23900 |  |  | “Greyzone” |  |
| 116 | Female | 39 | 253 | 17 | 14 | 0,14 | 29000 | 5,4 | Fatty liver | “Greyzone” |  |
| 117 | Male | 35 | 151 | 50 | 34 | 0,56 | 2120 | 4,9 | Unspecific liver pathology | Active hepatitis |  |
| 118 | Female | 18 | 283 | 16 | 20 | 0,18 | 18700 | 3,7 | Normal | “Greyzone” |  |
| 119 | Female | 45 | 336 | 23 | 35 | 0,26 | 8260 |  | Normal | “Greyzone” |  |
| 120 | Female | 26 | 301 | 41 | 42 | 0,35 | 11000 |  | Normal | Active hepatitis |  |
| 121 | Male | 30 | 186 | 16 | 20 | 0,27 | 577 | 6,4 | Normal | Low-replicative infection |  |
| 122 | Female | 40 | 215 | 20 | 22 | 0,26 | 166 |  |  | Low-replicative infection |  |
| 123 | Male | 18 | 111 | 471 | 625 | 14,08 | 5,04E+08 | 11,9 | Hepatomegaly | Active hepatitis |  |
| 124 | Female | 28 | 357 | 29 | 23 | 0,16 | 630 |  |  | Low-replicative infection |  |
| 125 | Female | 38 | 177 | 18 | 17 | 0,24 | 10 | 4,2 |  | Low-replicative infection |  |
| 126 | Male | 35 | 191 | 104 | 134 | 1,75 | 5860 |  | Unspecific liver pathology | Active hepatitis |  |
| 127 | Male | 38 | 324 | 34 | 30 | 0,23 | 305 |  | Normal | Low-replicative infection |  |
| 128 | Female | 52 | 260 | 16 | 23 | 0,22 | 2140 |  | Normal | “Greyzone” |  |
| 129 | Female | 22 | 287 | 14 | 18 | 0,16 | 11 |  | Normal | Low-replicative infection |  |
| 130 | Female | 24 | 220 | 17 | 23 | 0,26 | 2340 |  |  | “Greyzone” |  |
| 131 | Female | 26 | 172 | 43 | 58 | 0,84 | 31500 | 11,4 |  | Active hepatitis |  |
| 132 | Male | 34 | 123 | 76 | 400 | 8,13 | 0 |  | Cirrhosis, ascites | Cirrhosis-decomp |  |
| 133 | Female | 38 | 173 | 55 | 42 | 0,61 | 2010000 | 8,7 | Unspecific liver pathology | Active hepatitis |  |
| 134 | Male | 56 | 403 | 14 | 22 | 0,14 | 47 |  |  | Low-replicative infection |  |
| 135 | Male | 27 | 228 | 85 | 56 | 0,61 | 57900000 |  | Unspecific liver pathology | Active hepatitis |  |
| 136 | Male | 38 | 71 | 21 | 34 | 1,20 | 258 |  | Cirrhosis, PHT, splenomegaly | Cirrhosis - decomp |  |
| 137 | Male | 28 | 190 | 84 | 33 | 0,43 | 19600 | 4,5 | Normal | Active hepatitis |  |
| 138 | Male | 20 | 301 | 89 | 50 | 0,42 | 21600 | 4,3 |  | Active hepatitis |  |
| 139 | Male | 27 | 266 | 41 | 33 | 0,31 | 3670 | 4,6 | Normal | Active hepatitis |  |
| 140 | Female | 50 | 393 | 78 | 36 | 0,23 | 2270 |  | Normal | Active hepatitis |  |
| 141 | Female | 30 | 189 | 17 | 15 | 0,20 | 10 |  |  | Low-replicative infection | Positive |
| 142 | Male | 26 | 280 | 212 | 75 | 0,67 | 28700 | 5,4 |  | Active hepatitis |  |
| 143 | Male | 19 | 52 | 76 | 45 | 2,16 | 79 |  | Cirrhosis, PHT | Cirrhosis |  |
| 144 | Female | 25 | 260 | 70 | 25 | 0,24 | 32600 |  | Normal | Active hepatitis |  |
| 145 | Female | 55 | 148 | 35 | 51 | 0,86 | 1440 | 17,8 | Normal | Cirrhosis | Positive |
| 146 | Female | 27 | 189 | 26 | 20 | 0,26 | 7280 | 4 | Normal | “Greyzone” |  |
| 147 | Male | 45 | 38 | 43 | 60 | 3,95 | 1120 | 17,1 | Cirrhosis, ascites, PHT, splenomegaly | Cirrhosis - decomp |  |
| 148 | Female | 22 | 294 | 21 | 17 | 0,14 | 2000 |  |  | Low-replicative infection |  |
| 149 | Male | 36 | 106 | 16 | 22 | 0,52 | 18600000 | 16,6 | Cirrhosis, ascites, PHT | Cirrhosis - decomp |  |
| 150 | Male | 25 | 81 | 84 | 65 | 2,01 | 28700000 |  | Unspecific liver pathology | Active hepatitis |  |
| 151 | Male | 32 | 218 | 13 | 15 | 0,17 | 2990 | 4,8 | Normal | “Greyzone” |  |
| 152 | Female | 21 | 239 | 123 | 71 | 0,74 | 503000 | 6,8 | Normal | Active hepatitis |  |
| 153 | Male | 18 | 58 | 108 | 140 | 6,03 | 4700000 |  | Unspecific liver pathology | Cirrhosis - decomp |  |
| 154 | Female | 32 | 257 | 14 | 21 | 0,20 | 1600 | 4,1 |  | Low-replicative infection |  |
| 155 | Male | 38 | 234 | 23 | 20 | 0,21 | 8280 |  |  | “Greyzone” |  |
| 156 | Male | 33 | 191 | 18 | 21 | 0,27 | 129 | 3,4 | Normal | Low-replicative infection |  |
| 157 | Male | 27 | 230 | 16 | 22 | 0,24 | 1670 |  |  | Low-replicative infection |  |
| 158 | Male | 32 | 321 | 28 | 26 | 0,20 | 237 |  |  | Low-replicative infection |  |
| 159 | Female | 30 | 219 | 33 | 23 | 0,26 | 3790 | 4,8 |  | “Greyzone” |  |
| 160 | Male | 29 | 251 | 30 | 20 | 0,20 | 4890 | 4,8 | Normal | “Greyzone” |  |
| 161 | Female | 30 | 220 | 22 | 23 | 0,26 | 61 | 4,7 | Normal | Low-replicative infection |  |
| 162 | Male | 38 | 112 | 121 | 184 | 4,11 | 8660000 |  | Cirrhosis | Cirrhosis |  |
| 163 | Male | 28 | 274 | 20 | 24 | 0,22 | 228 | 4,6 | Normal | Low-replicative infection |  |
| 164 | Female | 45 | 207 | 15 | 20 | 0,24 | 549 |  | Normal | Low-replicative infection |  |
| 165 | Female | 22 | 248 | 21 | 26 | 0,26 | 93400 | 4 | Normal | “Greyzone” |  |
| 166 | Female | 28 | 309 | 11 | 17 | 0,14 | 1920 |  |  | Low-replicative infection |  |
| 167 | Male | 38 | 212 | 12 | 11 | 0,13 | 791 | 4,3 | Fatty liver | Low-replicative infection |  |
| 168 | Male | 18 | 261 | 43 | 46 | 0,44 | 50400000 | 6,8 | Normal | Active hepatitis |  |
| 169 | Male | 30 | 259 | 68 | 26 | 0,25 | 23800 |  | Unspecific liver pathology | Active hepatitis |  |
| 170 | Female | 50 | 219 | 25 | 23 | 0,26 | 219 |  | Normal | Low-replicative infection |  |
| 171 | Female | 29 | 161 | 52 | 31 | 0,48 | 4930 | 5,3 | Normal | Active hepatitis |  |
| 172 | Male | 40 | 220 | 8 | 13 | 0,15 | 1100 |  |  | Low-replicative infection |  |
| 173 | Female | 45 | 259 | 9 | 18 | 0,17 | 0 |  |  | Low-replicative infection |  |
| 174 | Female | 25 | 578 | 19 | 26 | 0,11 | 26700 | 4,8 |  | “Greyzone” |  |
| 175 | Female | 28 | 273 | 13 | 21 | 0,19 | 219 | 6 |  | Low-replicative infection |  |
| 176 | Female | 27 | 356 | 17 | 14 | 0,10 | 7480 |  | Normal | “Greyzone” |  |
| 177 | Female | 21 | 201 | 14 | 19 | 0,24 | 968 |  |  | Low-replicative infection |  |
| 178 | Male | 22 | 1404 | 56 | 31 | 0,06 | 3580 |  | Normal | Active hepatitis |  |
| 179 | Female | 28 | 173 | 11 | 17 | 0,25 | 333 |  |  | Low-replicative infection |  |
| 180 | Male | 22 | 222 | 56 | 57 | 0,64 | 14800 |  | Hepatomegaly | Active hepatitis |  |
| 181 | Male | 32 | 231 | 15 | 20 | 0,22 | 831 |  |  | Low-replicative infection |  |
| 182 | Female | 35 | 275 | 14 | 16 | 0,15 | 48 | 3,7 | Normal | Low-replicative infection |  |
| 183 | Male | 28 | 325 | 30 | 34 | 0,26 | 5660 | 5,5 | Unspecific liver pathology | “Greyzone” |  |
| 184 | Female | 21 | 297 | 15 | 15 | 0,13 | 10600 | 5,6 | Normal | “Greyzone” |  |
| 185 | Male | 50 |  | 18 | 35 |  | 21300 |  | Cirrhosis | Cirrhosis |  |
| 186 | Male | 35 | 235 | 13 | 17 | 0,18 | 667 | 4,7 | Hepatomegaly, splenomegaly | Low-replicative infection |  |
| 187 | Male | 43 | 138 | 19 | 23 | 0,42 | 1270 | 8,9 | Cirrhosis, PHT, splenomegaly | Cirrhosis |  |
| 188 | Female | 28 | 263 | 70 | 72 | 0,68 | 245000 | 5,1 | Normal | Active hepatitis |  |
| 189 | Male | 44 | 274 | 63 | 25 | 0,23 | 1010 | 3,9 | Cirrhosis | Cirrhosis |  |
| 190 | Female | 30 | 256 | 23 | 21 | 0,21 | 600 | 4,8 | Normal | Low-replicative infection |  |
| 191 | Male | 34 | 93 | 89 | 118 | 3,17 | 199000 | 20,6 | Cirrhosis, ascites | Cirrhosis - decomp |  |
| 192 | Female | 37 | 206 | 64 | 26 | 0,32 | 853 |  | Cirrhosis | Cirrhosis |  |
| 193 | Male | 20 | 266 | 43 | 94 | 0,88 | 6270 |  |  | Active hepatitis |  |
| 194 | Female | 29 | 262 | 26 | 19 | 0,18 | 4850 | 6,3 | Normal | “Greyzone” |  |
| 195 | Female | 19 | 384 | 481 | 1123 | 7,31 | 38900 | 5,3 | Unspecific liver pathology | Active hepatitis |  |
| 196 | Male | 40 | 93 | 31 | 49 | 1,32 | 9090000 |  | Cirrhosis, PHT | Cirrhosis - decomp |  |
| 197 | Male | 30 | 198 | 46 | 64 | 0,81 | 119000 |  | Normal | Active hepatitis |  |
| 198 | Male | 20 | 58 | 51 | 17 | 0,73 | 74700000 |  | Cirrhosis, splenomegaly | Cirrhosis - decomp |  |
| 199 | Male | 35 | 94 | 31 | 91 | 2,42 | 502000 |  | Cirrhosis, ascites, PHT, splenomegaly | Cirrhosis - decomp |  |
| 200 | Female | 36 | 269 | 19 | 17 | 0,16 | 10 | 4,8 | Normal | Low-replicative infection |  |
| 201 | Female | 28 | 328 | 23 | 18 | 0,14 | 78 | 4,8 |  | Low-replicative infection |  |
| 202 | Male | 36 | 117 | 196 | 311 | 6,65 | 19600000 |  | Unspecific liver pathology | Active hepatitis |  |
| 203 | Female | 18 | 387 | 37 | 37 | 0,24 | 485 |  |  | Low-replicative infection |  |
| 204 | Male | 27 | 189 | 56 | 62 | 0,82 | 1060000 | 12,1 |  | Cirrhosis |  |
| 205 | Male | 24 | 139 | 21 | 32 | 0,58 | 24100 | 9,3 | Cirrhosis | Cirrhosis |  |
| 206 | Female | 30 | 60 | 67 | 134 | 5,58 | 2E+08 | 17,1 |  | Cirrhosis |  |
| 207 | Female | 36 | 61 | 58 | 54 | 2,21 | 7050 | 17,3 |  | Cirrhosis |  |
| 208 | Female | 25 | 207 | 11 | 20 | 0,24 | 335 |  | Normal | Low-replicative infection |  |
| 209 | Male | 26 | 89 | 25 | 29 | 0,81 | 124000 |  | Cirrhosis | Cirrhosis |  |
| 210 | Female | 21 | 208 | 16 | 15 | 0,18 | 134 |  |  | Low-replicative infection |  |
| 211 | Female | 18 | 134 | 396 | 128 | 2,39 | 12700 |  | Fatty liver | Active hepatitis |  |
| 212 | Male | 20 | 96 | 30 | 34 | 0,89 | 36000 | 8,2 | Cirrhosis, PHT | Cirrhosis |  |
| 213 | Male | 23 | 146 | 58 | 46 | 0,79 | 452000 | 17,3 |  | Cirrhosis |  |
| 214 | Male | 26 | 34 | 38 | 35 | 2,57 | 407 | 14,5 | Cirrhosis, ascites | Cirrhosis - decomp |  |
| 215 | Female | 28 | 348 | 18 | 24 | 0,17 | 12200 | 4,7 |  | “Greyzone” |  |
| 216 | Female | 22 | 384 | 17 | 29 | 0,19 | 127 |  |  | Low-replicative infection |  |
| 217 | Male | 24 | 201 | 22 | 20 | 0,25 | 24 |  |  | Low-replicative infection |  |
| 218 | Male | 20 | 295 | 30 | 32 | 0,27 | 3560 | 8,6 | Normal | “Greyzone” |  |
| 219 | Male | 40 | 308 | 47 | 45 | 0,37 | 4480000 |  | Cirrhosis, ascites, PHT | Cirrhosis - decomp |  |
| 220 | Male | 75 | 254 | 22 | 14 | 0,14 | 66 |  | Normal | Low-replicative infection |  |
| 221 | Male | 35 | 174 | 33 | 14 | 0,20 | 364 |  | Normal | Low-replicative infection |  |
| 222 | Female | 43 | 254 | 23 | 26 | 0,26 | 74 |  |  | Low-replicative infection |  |
| 223 | Female | 35 | 284 | 17 | 29 | 0,26 | 5000 |  | Hepatomegaly | “Greyzone” |  |
| 224 | Male | 35 | 271 | 24 | 26 | 0,24 | 4160 | 6,6 | Normal | “Greyzone” |  |
| 225 | Female | 31 | 166 | 29 | 18 | 0,27 | 1810 | 4,9 |  | Low-replicative infection |  |
| 226 | Female | 23 | 422 | 28 | 46 | 0,27 | 10 |  | Normal | Low-replicative infection |  |
| 227 | Female | 20 | 304 | 14 | 31 | 0,25 | 55 |  | Normal | Low-replicative infection |  |
| 228 | Male | 30 | 205 | 37 | 61 | 0,74 | 3940 | 5,5 | Cirrhosis, PHT | Cirrhosis |  |
| 229 | Male | 30 | 115 | 14 | 4 | 0,09 | 893 | 4,5 | Normal | Low-replicative infection |  |
| 230 | Male | 28 | 91 | 68 | 113 | 3,10 | 2,04E+08 |  | Cirrhosis, ascites | Cirrhosis - decomp |  |
| 231 | Male | 38 | 244 | 16 | 4 | 0,04 | 61 | 3,4 |  | Low-replicative infection | Positive |
| 232 | Male | 22 | 83 | 90 | 116 | 3,49 | 69800000 |  |  | Active hepatitis |  |
| 233 | Female | 30 | 290 | 43 | 40 | 0,34 | 2770000 |  |  | Active hepatitis |  |
| 234 | Male | 50 | 269 | 15 | 25 | 0,23 | 256 |  |  | Low-replicative infection |  |
| 235 | Female | 24 | 292 | 17 | 19 | 0,16 | 33 |  |  | Low-replicative infection |  |
| 236 | Female | 26 | 237 | 11 | 15 | 0,16 | 289 | 3,7 |  | Low-replicative infection |  |
| 237 | Male | 25 | 198 | 73 | 65 | 0,82 | 7170000 | 11,8 | Cirrhosis | Cirrhosis |  |
| 238 | Female | 32 | 126 | 143 | 133 | 2,64 | 2110000 |  | Cirrhosis | Cirrhosis |  |
| 239 | Female | 29 | 209 | 22 | 18 | 0,22 | 931 | 4,9 |  | Low-replicative infection |  |
| 240 | Male | 30 | 116 | 43 | 40 | 0,86 | 206000 | 18,8 | Cirrhosis, splenomegaly | Cirrhosis |  |
| 241 | Male | 22 | 329 | 278 | 307 | 2,33 | 92400 | 6,6 | Normal | Active hepatitis |  |
| 242 | Female | 50 | 46 | 35 | 48 | 2,61 | 34800000 | 14,3 | Cirrhosis | Cirrhosis |  |
| 243 | Female | 28 | 244 | 21 | 17 | 0,17 | 12 |  | Normal | Low-replicative infection |  |
| 244 | Female | 21 | 180 | 75 | 53 | 0,74 | 2,37E+08 |  |  | Active hepatitis |  |
| 245 | Male | 45 | 212 | 21 | 23 | 0,27 | 18 |  |  | Low-replicative infection |  |
| 246 | Male | 43 | 249 | 31 | 26 | 0,26 | 61 | 4,1 | Normal | Low-replicative infection |  |
| 247 | Male | 38 | 121 | 50 | 71 | 1,47 | 19600000 |  | Cirrhosis, ascites, splenomegaly | Cirrhosis - decomp |  |
| 248 | Female | 29 | 240 | 18 | 25 | 0,26 | 12700 | 4,9 | Normal | “Greyzone” |  |
| 249 | Female | 26 | 175 | 22 | 18 | 0,26 | 196000 | 6,3 | Normal | “Greyzone” |  |
| 250 | Female | 25 | 286 | 10 | 20 | 0,17 | 2740 | 5,6 |  | “Greyzone” |  |
| 251 | Male | 51 | 317 | 22 | 24 | 0,19 | 388 | 3,7 | Normal | Low-replicative infection | Positive |
| 252 | Female | 38 | 227 | 5 | 21 | 0,23 | 534 |  | Normal | Low-replicative infection |  |
| 253 | Female | 22 | 60 | 17 | 36 | 1,50 | 74 |  | Cirrhosis, ascites, PHT splenomegaly | Cirrhosis - decomp |  |
| 254 | Female | 25 | 158 | 19 | 15 | 0,24 | 25600 | 4,9 | Normal | “Greyzone” |  |
| 255 | Male | 46 | 157 | 48 | 78 | 1,24 | 3370000 |  | Cirrhosis, PHT, splenomegaly | Cirrhosis - decomp |  |
| 256 | Female | 30 | 301 | 30 | 25 | 0,21 | 583 | 6 | Normal | Low-replicative infection |  |
| 257 | Male | 26 | 427 | 49 | 65 | 0,38 | 27200000 |  | Cirrhosis | Cirrhosis |  |
| 258 | Male | 27 | 257 | 14 | 22 | 0,21 | 10 |  | Normal | Low-replicative infection |  |
| 259 | Male | 53 | 199 | 23 | 15 | 0,19 | 157 |  | Normal | Low-replicative infection |  |
| 260 | Male | 29 | 800 | 40 | 21 | 0,07 | 3160 |  | Normal | “Greyzone” |  |
| 261 | Female | 36 | 236 | 14 | 21 | 0,22 | 11200 | 7,5 | Normal | “Greyzone” |  |
| 262 | Male | 30 | 276 | 131 | 132 | 1,20 | 39700000 | 6,6 | Unspecific liver pathology | Active hepatitis |  |
| 263 | Male | 27 | 241 | 14 | 13 | 0,13 | 40300 | 5,5 |  | “Greyzone” |  |
| 264 | Male | 36 | 384 | 15 | 17 | 0,11 | 607 |  | Cirrhosis, ascites, splenomegaly | Cirrhosis - decomp |  |
| 265 | Male | 36 | 232 | 11 | 16 | 0,17 | 166 | 4,8 | Normal | Low-replicative infection |  |
| 266 | Male | 25 | 277 | 27 | 21 | 0,19 | 2320 |  | Normal | “Greyzone” |  |
| 267 | Male | 21 | 193 | 14 | 15 | 0,19 | 84400 |  | Normal | “Greyzone” |  |
| 268 | Male | 30 | 4 | 60 | 91 | 56,88 | 14500 |  |  | Active hepatitis | Positive |
| 269 | Female | 26 | 251 | 46 | 34 | 0,34 | 13 | 5,6 | Cirrhosis | Cirrhosis |  |
| 270 | Female | 30 | 246 | 17 | 12 | 0,12 | 101 | 3,9 | Normal | Low-replicative infection |  |
| 271 | Male | 38 | 247 | 27 | 24 | 0,24 | 1740 |  | Normal | Low-replicative infection |  |
| 272 | Male | 40 | 262 | 119 | 152 | 1,45 | 290000 |  | Cirrhosis, ascites, HCC, portal vein thrombosis | Cirrhosis - decomp |  |
| 273 | Male | 28 | 199 | 30 | 20 | 0,25 | 48300 |  | Normal | “Greyzone” |  |
| 274 | Male | 31 | 106 | 42 | 38 | 0,90 | 3100 | 2,8 | Normal | Active hepatitis |  |
| 275 | Female | 20 | 314 | 147 | 86 | 0,68 | 11200000 |  | Normal | Active hepatitis |  |
| 276 | Female | 22 | 376 | 9 | 15 | 0,10 | 46 | 4,9 |  | Low-replicative infection |  |
| 277 | Male | 23 | 199 | 62 | 30 | 0,38 | 1E+09 | 4,9 | Cirrhosis | Cirrhosis |  |
| 278 | Male | 60 | 203 | 8 | 21 | 0,26 | 274 | 4 | Normal | Low-replicative infection |  |
| 279 | Female | 56 | 318 | 22 | 24 | 0,19 | 173 |  | Normal | Low-replicative infection |  |
| 280 | Female | 30 | 323 | 18 | 22 | 0,17 | 1460 | 3 |  | Low-replicative infection |  |
| 281 | Male | 28 | 9 | 23 | 37 | 10,28 | 396000 |  | Cirrhosis, ascites, PHT, splenomegaly | Cirrhosis - decomp |  |
| 282 | Female | 26 | 289 | 10 | 13 | 0,11 | 340 | 4,5 |  | Low-replicative infection |  |
| 283 | Female | 39 | 249 | 20 | 22 | 0,22 | 347 | 4,6 | Normal | Low-replicative infection |  |
| 284 | Male | 42 | 187 | 57 | 35 | 0,47 | 1370000 |  |  | Active hepatitis |  |
| 285 | Male | 40 | 78 | 86 | 119 | 3,81 | 254000 |  | Cirrhosis, ascites, splenomegaly | Cirrhosis - decomp |  |
| 286 | Female | 20 | 198 | 14 | 20 | 0,25 | 5080 |  | Normal | “Greyzone” |  |
| 287 | Male | 30 | 193 | 24 | 21 | 0,27 | 170000 | 5,7 | CLD | “Greyzone” |  |

**Table S1**

Patient characteristics including sex, age, platelets, ALT, AST, APRI score, HBV DNA, LSM (kPa), ultrasound findings, patient category and hepatitis D status (if positive). ALT; alanine transaminase, AST; aspartate aminotransferase, APRI; AST to platelet ratio, HBV; hepatitis B virus, LSM; liver stiffness measurement, PHT; portal hypertension.

**Table S2**

| **Name** | **Supplier** | **Cat no.** | **Clone no.** |
| --- | --- | --- | --- |
| Mouse anti-human CD127 FITC | Biolegend | 351311  RRID: AB_10897643 | A019D5 |
| Mouse anti-human CD141 BB630 | BD biosciences | Custom antibody | 1A4 |
| Mouse anti-human CD1c BB700 | BD biosciences | 746095  RRID: AB_2743468 | F10/21A3 |
| Mouse anti-human CD16 BB755 | BD biosciences | Custom antibody | 3G8 |
| Mouse anti-human CD38 BB790 | BD biosciences | Custom antibody | HIT2 |
| Mouse anti-humam IgD APC | Biolegend | 348222  RRID: AB_2561595 | IA6-2 |
| Mouse anti-human CD45 AF700 | Biolegend | 304024  RRID: AB_493761 | HI30 |
| Mouse anti-human PD-1 BV421 | Biolegend | 329920  RRID: AB_10960742 | EH12.2H7 |
| Mouse anti-human CD4 BV510 | Biolegend | 317444  RRID: AB_2561866 | OKT4 |
| Mouse anti-human IgM BV570 | Biolegend | 314518  RRID:  AB_10913816 | MHM-88 |
| Mouse anti-human KLRG1 BV605 | BD bioscience | 568891  RRID:  AB_2563357 | Z7-205.rMAb |
| Mouse anti-human CD161 BV650 | BD bioscience | 563864  RRID:  AB_2738456 | DX12 |
| Mouse anti-human CD25 BV711 | BD bioscience | 563159  RRID:  AB_2738037 | 2A3 |
| Mouse anti-human CD15 BV750 | BD bioscience | 747426  RRID:  AB_2872112 | W6D3 |
| Mouse anti-human CD19 BV786 | BD bioscience | 563325  RRID:  AB_2744314 | SJ25C1 |
| Mouse anti-human HLA-DR BUV395 | BD bioscience | 564040  RRID: AB_2738558 | G46-6 |
| Mouse anti-human CD8 BUV496 | BD bioscience | 612942  RRID:  AB_2870223 | RPA-T8 |
| Mouse anti-human CD56 BUV563 | BD bioscience | 612928  RRID:  AB_2870213 | NCAM16.2 |
| Mouse anti-human CD20 BUV615-P | BD bioscience | 751256  RRID:  AB_2875272 | 2H7 |
| Mouse anti-human CD3 BUV661 | BD bioscience | 612964  RRID:  AB_2869644 | UCHT1 |
| Mouse anti-human CD45RA BUV737 | BD bioscience | 612846  RRID:  AB_2870168 | HI100 |
| Mouse anti-human CD45 BUV805 | BD bioscience | 612891  RRID: AB_2870179 | HI30 |
| Mouse anti-human CCR7 PE | Biolegend | 353204  RRID:  AB_10913813 | G043H7 |
| Mouse anti-human TCRVa7.2 PE-Dzl594 | Biolegend | 351730  RRID: AB_2566339 | 3C10 |
| Mouse anti-human CD27 PC5.5 | Beckman Coulter | B21444  RRID: AB_2934286 | 1A4CD27 |
| Mouse anti-human IgA PE-Vio770 | Miltenyi | 130-113-477 RRID: AB_2751114 | IS11-8E10 |
| Mouse anti-human CD39 BB515 | BD bioscience | 565469  RRID: AB_2722754 | TU66 |
| Mouse anti-human BB630 CXCR5 | BD bioscience | Custom antibody | RF8B2 |
| Mouse anti-human CD57 BB660 | BD bioscience | Custom antibody | HNK-1 |
| Mouse anti-human TIM-3 BB700 | BD bioscience | 746178  RRID:  AB_2871643 | 7D3 |
| Mouse anti-human CD122 APC | Biolegend | 339008  RRID: AB_2123575 | TU27 |
| Mouse anti-human PD-1 APCFire750 | Biolegend | 329954  RRID: AB_2616721 | EH12.2H7 |
| Mouse anti-human CX3CR1 BV570 | Biolegend | 341634  RRID: AB_3068294 | 2A9-1 |
| Mouse anti-human  NKp46 BV650 | Biolegend | 331927  RRID: AB_2562442 | 9E2 |
| Mouse anti-human CD62L BV750 | BD bioscience | 747422  RRID: AB_2872109 | DREG-56 |
| Mouse anti-human CD69 BV786 | BD bioscience | 563834  RRID:  AB_2738441 | FN50 |
| Mouse anti-human NKG2D BUV615-P | BD bioscience | 751232  RRID: AB_2875253 | 1D11 |
| Mouse anti-human CD134 (OX40) PE-Dzl594 | Biolegend | 350020  RRID:  AB_2571940 | Ber-ACT35 |
| Mouse anti-human CD14 PE-Cy5 | Thermo Fisher | 15-0149-42  RRID:  AB_2573058 | 61D3 |
| Mouse anti-human CD19 PE-Cy5 | Biolegend | 302210  RRID: AB_314240 | HIB19 |
| Mouse anti-human NKG2A PE-Vio770 | Miltenyi | 130-113-567 RRID:  AB_2726172 | REA110 |
| Mouse anti-human CD14 APC-Cy7 | Biolegend | 301820  RRID:  AB_493695 | M5E2 |

Table S2 List of antibodies with supplier, catalogue number and clone.

**Table S3**

| **Immune cell** | **Gating hierarchy panel 1** |
| --- | --- |
| NKT cell | CD15-, CD45+, CD3+, CD56+ |
| MAIT cell | CD15-, CD45+, CD3+, CD161+, TCRVα7.2+ |
| CD8+ T cell | CD15-, CD45+, CD3+, CD8+ |
| Naïve CD8+ T cell | CD15-, CD45+, CD3+, CD8+, CD45RA+, CCR7+ |
| Central memory CD8+ T cell | CD15-, CD45+, CD3+, CD8+, CD45RA-, CCR7+ |
| Effector memory CD8+ T cell | CD15-, CD45+, CD3+, CD8+, CD45RA-, CCR7- |
| Terminal effector memory (TEMRA) CD8+T cell | CD15-, CD45+, CD3+, CD8+, CD45RA+, CCR7- |
| CD4+ T cell | CD15-, CD45+, CD3+, CD8-, CD4+ |
| CD4+ regulatory T cell | CD15-, CD45+, CD3+, CD8-, CD4+, CD25+, CD127- |
| Naïve CD4+ T cell | CD15-, CD45+, CD3+, CD8-, CD4+, CD25-, CD45RA+, CCR7+ |
| Central memory CD4+ T cell | CD15-, CD45+, CD3+, CD8-, CD4+, CD25-, CD45RA-, CCR7+ |
| Effector memory CD4+ T cell | CD15-, CD45+, CD3+, CD8-, CD4+, CD25-, CD45RA-, CCR7- |
| Terminal effector memory CD4+ T cell | CD15-, CD45+, CD3+, CD8-, CD4+, CD25-, CD45RA+, CCR7- |
| NK cell | CD15-, CD45+, CD3-, CD19-, CD56+ |
| CD56^bright^ NK cell | CD15-, CD45+, CD3-, CD19-, CD56++, CD16+ |
| CD56^dim^ NK cell | CD15-, CD45+, CD3-, CD19-, CD56+, CD16++ |
| CD141+ Dendritic cell | CD15-, CD45+, CD3-, CD19-, CD56-, HLA-DR+, CD141+ |
| CD1c+ Dendritic cell | CD15-, CD45+, CD3-, CD19-, CD56-, HLA-DR+, CD1c+ |
| Monocyte | CD15-, CD45+, CD3-, CD19-, CD56-, HLA-DR+, CD141-, CD1c- |
| Classical monocyte | CD15-, CD45+, CD3-, CD19-, CD56-, HLA-DR+, CD141-, CD1c-, CD14++, CD16- |
| Intermediate monocyte | CD15-, CD45+, CD3-, CD19-, CD56-, HLA-DR+, CD141-, CD1c-, CD14+, CD16+ |
| Non-classical monocyte | CD15-, CD45+, CD3-, CD19-, CD56-, HLA-DR+, CD141-, CD1c-, CD14-, CD16++ |
| B cell | CD15-, CD45+, CD3-, CD19+ |
| Plasma cells | CD15-, CD45+, CD3-, CD19+, CD38+, CD20-, CD27+ |
| Precursors | CD15-, CD45+, CD3-, CD19+, CD38+, CD20-, CD27- |
| Naive B cell | CD15-, CD45+, CD3-, CD19+, CD20+, IgD+, CD27- |
| Atypical memory B cell | CD15-, CD45+, CD3-, CD19+, CD20+, IgD-, CD27- |
| Memory B cell | CD15-, CD45+, CD3-, CD19+, CD20+, IgD-, CD27+ |
| Unswitched memory B cell | CD15-, CD45+, CD3-, CD19+, CD20+, IgD+, CD27+ |

Table S3 Hierarchical gating strategy for all major immune cell subtypes.

**Table S**4

| **Immune cell subtype** | **Low-replicative infection** | **Greyzone** | **Active hepatitis** | **Cirrhosis** |
| --- | --- | --- | --- | --- |
| CD8+ T cells (cells/µL) | 255 [152–521] | 215 [128–463] | 206 [132–343] | 118 [63–270] |
| CD4+ T cells (cells/µL) | 470 [269–803] | 386 [255–809] | 473 [273–705] | 209 [120–408] |
| Tregs (cells/µL) | 7.9 [2.7–13.3] | 5.7 [3.1–10.2] | 6.1 [2.3–14.2] | 3.3 [1.1–6.4] |
| NK cells (cells/µL) | 61 [28–117] | 51 [24–87] | 62 [23–129] | 32 [12–77] |
| B cells (cells/µL) | 114 [59–223] | 121 [59–233] | 97 [52–181] | 70 [31–108] |
| Monocytes (cells/µL) | 165 [87–287] | 134 [73–198] | 168 [103–321] | 135 [69–277] |

Table S4 Median [interquartile range] absolute cell counts of major cell groups given as cells/µL for each clinical group.
